# Oscillatory signatures of monitoring and anticipatory strategies for probabilistic vs deterministic cues

**DOI:** 10.1162/imag_a_00496

**Published:** 2025-03-07

**Authors:** Luca Tarasi, Riccardo Bertaccini, Giuseppe Ippolito, Maria Eugenia Martelli, Giuseppe di Pellegrino, Vincenzo Romei

**Affiliations:** Dipartimento di Psicologia, Università di Bologna and Centro studi e ricerche in Neuroscienze Cognitive, Università di Bologna, Cesena, Italy; Universidad Antonio de Nebrija, Madrid, Spain

**Keywords:** predictive coding, prior knowledge, perceptual decision-making, inter-individual differences, theta oscillations, beta oscillations

## Abstract

Perception is not exclusively determined by sensory input, being strongly shaped by expectations. Here, we manipulated target occurrence certainty—random (50%), probabilistic (63/75%), deterministic (100%)—to investigate how priors shape decision-making. Results revealed strong influence of expectations on decision-bias, with modulation increasing as priors attain predictive power. This influence was particularly evident in deterministic trials, where the prior’s absolute validity heightened performance. Notably, individuals exhibited wide variability in predictive strategies: some exhibited strong prior-driven choice (*believers*), while others relied more on sensory input (*empiricists*). Relative to*empiricists*,*believers*exhibited reduced midfrontal theta rhythm in probabilistic trials, indicating less monitoring for actual target occurrence, and higher motor beta desynchronization in deterministic trials, suggesting a shift toward motor strategy implementing prior-congruent action. Crucially,*believers’*prior-driven approach conferred an advantage in deterministic conditions. These findings highlight priors’ impact on decision-making, emphasizing the interplay between monitoring and anticipatory mechanisms in leveraging expectations.

## 1 Introduction

According to the Bayesian brain framework (BBF), our decision stems from the integration of what our senses capture (i.e., sensory evidence) with our pre-existing expectations about the external world (i.e., prior model) (Clark, 2013). This theory has been substantiated by several empirical studies, showcasing how prior information significantly shapes response rate (Bang & Rahnev, 2017), reaction times (Mulder et al., 2012), metacognition (Sherman et al., 2015), and decisional criterion (Tarasi, di Pellegrino, et al., 2022;Wyart et al., 2012). A pivotal aspect in BBF revolves around the predictive power of prior information, as it directly determines the degree to which prior models exert influence over current perceptual experiences (Mathys et al., 2011). When prior expectations exhibit higher validity, the system accords them greater importance by greatly shaping the ongoing perception. Conversely, lower validity implies a system more amenable to adaptation based on newly acquired sensory information. Crucially, prior validity is shaped not only by the inherent predictive power of prior models—with a 90% validity indicating higher precision than one with a validity of 60%—but also by a subjective element tied to interindividual differences in prior weighting (Tarasi et al., 2023).

In a previous study, we have outlined that individuals were able to shift from a conservative strategy (i.e., a tendency to report the absence of the target) to a liberal strategy (i.e., a tendency to report the presence of the target) when an expectation cue signaled low versus high probability target presence (Tarasi, di Pellegrino, et al., 2022). Crucially, there was strong variability within the sample concerning the weighting of prior information (Tarasi, di Pellegrino, et al., 2022). This variability allowed us to identify two main decision-making strategies along a continuum. At one extreme is the strategy of the*believer*(an individual who tends to overuse prior information in the face of sensory input), while at the opposite end is the strategy of the*empiricist*(an individual who tends to underuse prior information, in favor of sensory input information).

In this study, we aimed to investigate the neurobehavioral markers underlying the parametric exploitation of expectation-like information employing an EEG protocol. Specifically, in the established protocol, prior validity ranged from random (50% target probability occurrence) to deterministic (100%), with intermediate levels characterized by mid (63%) or high validity (75%). Therefore, in contrast toTarasi, di Pellegrino, et al. (2022)andTarasi et al. (2025), which involved two contrasting priors (i.e., low probability target presence, 33% leading to more conservative decision strategies vs. high probability target presence, 66%, leading to more liberal decision strategies), the current protocol prompts the adoption of a single decision strategy, that is, the liberal one.

The possibility of employing a single graded strategy could trigger motor mechanisms that anticipate the prior-congruent movement. Indeed, expectations derived from prior probabilities could guide motor preparation by enabling participants to anticipate and pre-plan actions that align with the most likely outcome, thus optimizing decision-making processes (Kelly et al., 2021;Tarasi, Tabarelli de Fatis, et al., 2024). In the random condition, the feasibility of motor preparation is absent since both motor actions (i.e., movements signaling target presence vs. absence) are equiprobable. However, as prior predictive power increases, programming responses (i.e., indicating target presence) aligned with the prior-related strategy (i.e., liberal) become a viable option. This capacity would reach its peak in the deterministic condition, where it is possible to fully program the response even before the appearance of a stimulus. At the neurophysiological level, beta rhythms have been associated with motor preparation processes. Specifically, a desynchronization in the beta band has been observed both before and during voluntary movement, with the degree of desynchronization being influenced by prior information (Lange et al., 2013). Furthermore, activity in the beta range can predict subjects’ choices several seconds prior to overt manual responses (Donner et al., 2009), serving as an indicator of the probability that a new voluntary action will be initiated (Jenkinson & Brown, 2011). Therefore, we hypothesized the presence of a desynchronization in the beta band within the motor areas contralateral to the hand responsible for signaling the behavioral response. This desynchronization is expected to intensify with the increasing predictive power of the prior information (Qin et al., 2023), reaching its peak in the deterministic condition.

In addition, inTarasi, di Pellegrino, et al. (2022), we found that liberal trials were characterized by theta oscillatory activations which emerged as a candidate marker in governing the hypothesized monitoring mechanism on prior exploitation. Frontal theta oscillations have long been associated with a comprehensive mechanism governing the enactment of cognitive control (Cavanagh & Frank, 2014), and they have been involved in goal-directed, strategic, and adaptive behavior (Cavanagh et al., 2010;Cavanagh, Figueroa, et al., 2012). Moreover, theta rhythms emerge in response to novelty, conflict, errors, and negative feedback, highlighting their connection with situations requiring cognitive control (Cavanagh, Zambrano-Vazquez, et al., 2012). While early investigations primarily focused on the reactive role of theta rhythm (i.e., increase following the occurrence of cognitively challenging events), recent evidence suggests a proactive theta regulation. This proactive employment involves pre-setting the cognitive system to be responsive to demanding situations (Cooper et al., 2015), particularly when preparing to override a prepotent response tendency (van Noordt et al., 2017). For instance, an increase of theta in the frontal areas functions as an inhibitory mechanism, diminishing the impact of salient attributes in value-based decision-making, ultimately predicting regulatory success (HajiHosseini & Hutcherson, 2021). Conversely, the inability to implement the theta-based monitoring mechanism may lead to an inclination to rely more on automatic mechanisms. For instance, individuals exhibiting lower frontal theta power in response to Pavlovian conflict were found to be more susceptible to bias derived from Pavlovian acquisition (Cavanagh et al., 2013).

We anticipate that both the theta-based monitoring process and the beta-based motor preparation will be shaped by interindividual differences in prior handling. Specifically, we expect a reduction in midfrontal theta-based monitoring and a more pronounced anticipatory motor beta-desynchronization in individuals who extensively rely on prior information (*believers*), relative to individuals who tend to downplay prior information (*empiricists*). The latter are anticipated to be less characterized by anticipatory mechanisms, as the outcome of their choices about target presence relies on sensory elements extracted from stimuli, rather than predicting their presence based on prior information.

## 2 Methods

### 2.1 Participants

Fifty-four participants (30 female; age range 18–35) signed a written informed consent prior to taking part in the study, which was conducted in accordance with the Declaration of Helsinki and approved by the Bioethics Committee of the University of Bologna.

### 2.2 Stimuli

Stimuli were displayed on an 18’ CRT monitor (Cathode Ray Tube, CRT, with a display resolution of 1280 x 1024 pixels and a refresh rate of 85 Hz) at a distance of 57 cm in a dimly lit room. Participants were seated comfortably in front of the monitor. The stimuli, consisting of checkerboards presented in the lower left visual field, were generated and presented using Matlab (version 2016, The MathWorks Inc., Natick, MA) and the Psychophysics toolbox. The checkerboards could either contain grey circles within each cell (target) or not (catch trials). Participants were instructed to quickly and accurately indicate the presence (by pressing the “k” key with the middle finger) or absence (by pressing the “m” key with the index finger) of grey circles inside the checkerboard. Participants were specifically instructed to use their right hand for responses.

### 2.3 Experimental design

**Fig. 1.**
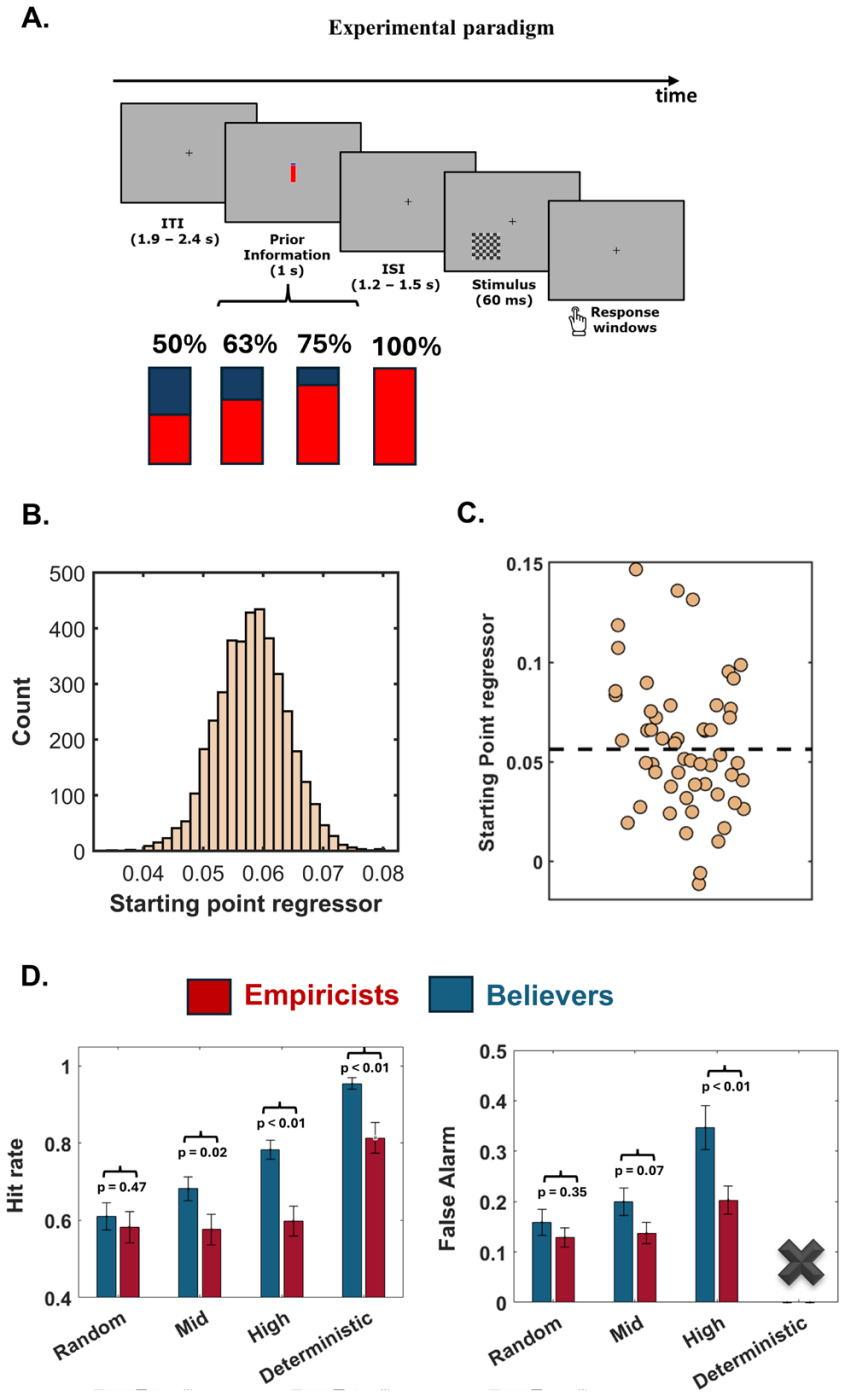
Experimental design and behavioral results. (A) EEG data were collected during a simple visual detection task. Each trial started with a fixation cross, after which a probabilistic cue appears in the center of the screen. After this, a checkerboard containing (or not) grey circles at the titrated contrast within it appeared at the bottom left of the monitor for 60 ms. The presented cues took the form of a blue bar, filled with different levels of red ink in the proportion of 50%, 63%, 75%, and 100% of the bar. The degree of red filling of the blue bar was related to the probability of target presence, with 63% indicating mid probability and 75% high probability of target presence, while the half-filled (50%) bar represented the random target probability occurrence, being thus uninformative of target presence versus absence. Finally, the fully filled (red) bar indicates the presence of the target in 100% of cases. (B) The starting point parameter was modulated by the validity assigned to the prior. By extracting credibility intervals, we showed that the starting point regressor has a positive coefficient that does not overlap with zero. This indicates that as the target presence prior validity increases, there is a corresponding shift in the starting point of accumulation toward the threshold signaling stimulus presence. (C) We represented the mean of the posterior distributions for each participant for the starting point regressor parameter. There are significant interindividual differences in the degree of starting point shift. As evident in the figure, some individuals have a regressor slope very close to zero (i.e., they do not shift the starting point based on the prior), while others exhibit highly positive values. We leveraged this variability to better characterize the decision-making strategies among participants through a median split analysis. This allowed us to extract the group of believers (who show a strong starting point shift) and the group of empiricists (who tend to accumulate sensory evidence starting from the same level without being heavily influenced by the prior). (D) Participants showed progressively higher hit rates as the prior gains predictive power. However, believers exhibited a greater increase compared to empiricists across all considered conditions. It is noteworthy that the initial hit rates (i.e., random trials) did not differ between the groups. The difference between the two performances reached its peak in the deterministic condition, where believers were very close to the maximum achievable threshold (i.e., 100%), reaching 97% correct target identifications compared to 81% of the empiricists. A similar increase is observed in false alarms, which rise as the prior becomes more precise. Again, individual decision-making style impacted this index, as participants within the believers group showed a significant increase in false alarms in the condition where the prior had high predictive power. It is noteworthy that it was not possible to calculate false alarms in the deterministic condition, as it consisted of 100% target-presence trials. Error bars represent the mean ± standard error of the mean.

The study was divided into two phases. In the first, each participant underwent an adaptive titration procedure to determine the contrast of the grey circles for which the detection accuracy was at ~70% when an equal number of target-present and target-absent trials (catch trials) was presented (for a detailed explanation of the procedure seeTarasi & Romei, 2024). The second phase comprised 6 blocks of 98 trials each (Fig. 1). Each trial started with the appearance of the probability cue presented at the center of the screen. The cue was presented for 1 s followed by a fixation dot. After a variable delay of 1.2–1.5 s, a checkerboard containing (or not) grey circles at the titrated contrast within it appeared at the bottom left of the monitor for 60 ms. We opted to present the stimulus in only one hemifield to prevent spontaneous fluctuations in attention between the two hemifields in the prestimulus period from interfering with the results. Participants had to determine the presence/absence of the grey circles within the checkerboard and press the button associated with their choice. No timeout has been set for the response. After collecting the response, the screen turned black for 1.9–2.4 s in the inter-trial interval. The cue consisted of a bar with its bottom colored in red and its top colored in blue. The percentage of the red shading to the entire bar indicated the probability that the checkerboard contained the grey circles (target) within it. Four cue levels were employed, each being associated with a distinct level of prior predictive power. In the random condition, the prior validity was fixed at 50%, signifying a lack of predictability for both target presence and absence. Mid- and high-validity priors indicated, respectively, 63% and 75% target presence probability. Additionally, deterministic prior accurately predicted the target’s presence with 100% validity. The actual target presentation probability was in accordance with the probability indicated by the cue. Participants were explicitly informed that the probabilistic cue accurately reflected the likelihood of the stimulus being presented.

### 2.4 Behavioral analysis

In order to characterize individuals depending on whether they show strong decisional modulations (i.e.,*believers*) versus minimal decisional modulations (i.e.,*empiricists*) based on priors, we fit participants’ decision using the drift diffusion model (DDM), the most widely used computational model of two-alternative decision-making tasks (Ratcliff et al., 2016;Wiecki et al., 2013). DDM takes simultaneously into consideration the accuracy of the choice and the response time distributions to estimate the decisional parameters (Supplementary Fig. S1). It conceptualizes decisions as an evidence accumulation process, where noisy information is integrated over time until it reaches a decision threshold. Among its key parameters are the drift rate, which reflects the speed and quality of evidence accumulation, and the starting point of the accumulation process. The starting point is particularly relevant for modeling the influence of priors, as it can shift toward one decision boundary based on expectations or prior information. This shift represents a decision bias, reflecting a predisposition to favor one response over another even before evidence is accumulated, thereby directly linking priors to decision-making behavior. Specifically, we estimated regression coefficients [*HDDMRegressor*(Wiecki et al., 2013)] to determine the relationship between the probabilistic prior type (3 levels: random, mid validity and high validity) and DDM parameters*z*(the starting point of the accumulation process). Higher starting points signal the accumulation process to shift more toward the threshold that codes for response presence. Please note that the deterministic prior condition could not be included in this analysis due to the absence of the stimulus-absent trials, which are mandatory for the model fitting procedures. We initialized HDDM to draw 5000 posterior samples with the first 1000 samples discarded as burn-in. We inspected traces of model parameters and their autocorrelation to ensure that the models had properly converged (Supplementary Fig. S2). Then, for each participant, a regression coefficient was extracted (*starting point regressor*), describing how much the cue was able to modulate the starting position of the accumulation process. High values implied a consistent shift of the starting point position, while reduced values suggested a reduced modulation of decisional bias based on prior validity. We conducted Bayesian statistics by computing the posterior distribution of the*starting point regressor*. We calculated credible intervals, which represent ranges of values where the true parameter value is likely to lie with a certain level of credibility (95%). Subsequently, we assessed whether the credible intervals overlap with zero. A significant difference from zero at the chosen confidence level would indicate that the predictive power of the prior significantly shapes the starting point parameter. To confirm that the prior affected only the starting point and not the rate of sensory information accumulation, we ran an additional HDDM model. In this analysis, we estimated regression coefficients to assess the relationship between the type of probabilistic prior and the DDM parameter v (the rate of evidence accumulation). This control analysis showed that the credible intervals of the resultant regressor overlapped with zero for 95% of its values, indicating that the prior did not impact evidence accumulation (Supplementary Fig. S3). Finally, we employed a median-split approach on the starting point regressors to categorize participants into two groups. This allowed tracing a group of individuals demonstrating a strong decisional modulation based on priors (i.e.,*believers*) versus a minimal modulation (i.e.,*empiricists*). It is worth emphasizing that, in our previous papers (Tarasi, di Pellegrino, et al., 2022;Tarasi et al., 2023), we distinguished between these two decision-making strategies by examining the change in alpha amplitude between low (i.e., 33% target probability) and high (i.e., 67% target probability) conditions. However, the modifications we made to the current experimental protocol, aimed at investigating the neurobehavioral processes underlying the adoption of a single-graded liberal strategy (with participants facing the question of “what is the degree of stimulus presence, ranging from 50% up to 100% occurrence?” rather than contrasting stimulus presence vs. absence), prevented us from using the same approach. This is because, while alpha shift consistently detects different strategies when a contrasting strategy (higher probability of stimulus presence vs. higher probability of stimulus absence) has to be implemented (Kloosterman et al., 2019), with higher versus lower synchronization in the low versus high condition, it is less sensitive to trace inter-individual differences in prior weighting when a single-grade strategy is to be enacted (seeSupplementary Materials). Consequently, in light of the different protocol we employed, we directly differentiate the two groups based on their actual behavioral performance.

To verify and further explore the division made, we used the raw hit rates and the raw false alarms as well as RTs to evaluate whether the behavioral indices extracted using different models confirmed the presence of two different predictive strategies. We anticipate that, if the division reliably captures the two predictive strategies, believers will demonstrate higher hit rates and false alarms as the prior validity increases. First, we computed the hit-rate (HR) and false alarm-rate (FA) in order to understand the impact of prior information on decisional outcomes for the two extracted groups. Hit-rates were computed for all cue conditions (i.e., random, mid-validity, high validity, and deterministic prior), while false alarm rates were calculated for the same conditions, except for the deterministic one, which lacked catch trials due to its nature (100% target-present trials). To statistically investigate a cue-related effect on hit rate, we ran a repeated-measures ANOVA, with the cue type as within-factors (4 levels: random, mid, high, deterministic) and the group as between-subject factor (2 levels: believers and empiricists). We conducted the very same analysis considering the FA, with the difference that the within-factors cue type had 3 levels (random, mid, high). Additionally, for each participant, we calculated the mean RTs for each type of response. For “present” responses, RTs were computed across the random, mid, high, and deterministic predictive value conditions. For “absent” responses, RTs were calculated for the random, mid, and high conditions, excluding the deterministic condition due to the limited or absent responses in this condition in some participants. Separate ANOVAs were then conducted for “present” and “absent” responses, with cue predictability and group as factors.

Moreover, we computed two additional bias indices: RT bias and Response bias. To calculate the RT bias, the following differences between RT in different conditions were used:

$$\text{RT bias index }=\text{ mean }\left(\begin{array}{l} {}[{\text{RT}}_{\text{mid},\text{present}}-{\text{RT}}_{\text{random},\text{present}}],\\ {}[{\text{RT}}_{\text{high},\text{present}}-{\text{RT}}_{\text{random},\text{present}}],\\ {}[{\text{RT}}_{\text{random},\text{absent}}–{\text{RT}}_{\text{mid},\text{absent}}],\\ \left[{\text{RT}}_{\text{random},\text{absent}}–{\text{RT}}_{\text{high},\text{absent}}\right] \end{array}\right)$$

A lower value for this index indicates a stronger bias in responding faster in prior-congruent responses.

For the Response bias index, we calculated the mean of the differences between hit rates and false alarms in the “mid”, “high”, and “random” conditions:

$$\text{Response bias index}=\text{mean }\left(\begin{array}{l} {}[{\text{HR}}_{\text{mid},\text{present}}-{\text{HR}}_{\text{random},\text{present}}],\\ {}[{\text{HR}}_{\text{high},\text{present}}-{\text{HR}}_{\text{random},\text{present}}],\\ {}[{\text{FA}}_{\text{random},\text{absent}}–{\text{FA}}_{\text{mid},\text{absent}}],\\ \left[{\text{FA}}_{\text{high},\text{absent}}–{\text{ FA}}_{\text{random},\text{abs}e\text{nt}}\right] \end{array}\right)$$

A higher value for this index indicates a greater decision bias, with participants more likely to commit both hits and false alarms as the target presence probability increases.

Finally, we calculated the criterion index using the Signal Detection Theory (Macmillan & Creelman, 1991), separately for the random, mid, and high conditions, where the criterion values reflects the participant’s decision bias toward responding “signal present” or “signal absent”. We then extracted the criterion shift by calculating the mean of the differences between the criterion in the mid and random conditions, and between the criterion in the high and random conditions.

### 2.5 EEG preprocessing and time frequency decomposition

Participants comfortably sat in a room with dimmed lights. A set of 64 electrodes was mounted according to the international 10–10 system. EEG signals were acquired at a rate of 1000 Hz, and all impedances were kept below 10 kΩ. EEG was processed offline with custom MATLAB scripts (version R2021a) and with the EEGLAB toolbox (Delorme & Makeig, 2004). The EEG recording was filtered offline in the 0.5–100 Hz band, and a notch-filter at 50 Hz was applied. The signals were visually inspected, and noisy channels were spherically interpolated. Epochs spanning −4100 to 3000 ms relative to checkerboard onset were extracted, and individual trials were visually checked and those containing excessive noise, muscle or ocular artefacts discarded. Next the recording was re-referenced to the average of all electrodes. We then applied the Independent Component Analysis (ICA), an effective method largely employed for removal of EEG artefacts. Components containing artifacts that could be clearly distinguished from brain-driven EEG signals were subtracted from the data. After these steps, we downsampled the signals to 256 Hz and a Laplacian transform was applied to the data using spherical splines. Subsequently, we implemented time-frequency analysis by convolving the time series data with a set of complex Morlet wavelets (whose cycles increased between three and eight cycles as a function of frequency), defined as complex sine waves tapered by a Gaussian distribution. Convolution was performed via frequency-domain multiplication, in which the Fourier-derived spectrum of the EEG data was multiplied by the spectrum of the wavelet, and the inverse Fourier transform was taken. Then, we obtained the amplitude by extracting the absolute value of the resulting complex time series. Amplitude was then condition-specific baseline-corrected using a decibel (dB) transform:$dB\;amplitude=\;10\;\times \;\log_{10}(amplitude\;/\;$ baseline)*.*Baseline amplitude was defined as the average amplitude in the period ranging from -3100 to -2700 ms before stimulus onset.

### 2.6 Oscillatory amplitude analysis: Fronto-central electrodes

To assess the monitoring impact of midfrontal theta on anticipatory cues, we focused the amplitude analysis on a cluster of fronto-central electrodes by averaging the amplitude of the following electrodes to avoid the potential issues associated with the selection of a restricted number of sensors: F1, FZ, F2, FC1, FCZ, FC2 (Alsuradi et al., 2021;Cooper et al., 2017;Jia et al., 2020;Ma et al., 2016;Pezzetta et al., 2018;Stolz et al., 2023;Wen et al., 2022;Wu et al., 2015;Züst et al., 2019). The rationale for this selection is based on substantial evidence indicating that frontal theta activity is most prominently recorded in the chosen electrodes. In order to specifically investigate the pre-stimulus oscillatory activity related to the prior use, a frequency per time nonparametric cluster-based permutation tests (n = 1.000) was performed on the amplitude difference between 1) the random- and mid-validity prior, 2) the random- and high-validity prior, and 3) the random- and determinist-prior in all time points ranging from -500 to 0 ms relative to the checkerboard appearance by shuffling the type of trials for each individual for each permutation in order to create a dummy distribution of amplitude difference. This method is data-driven and allows to test, point-by-point, the significant differences between the two types of prior information in the entire time interval considered and for all the frequencies included, controlling for multiple comparisons (Maris & Oostenveld, 2007).

Subsequently, we computed, for each individual, the mean amplitude value of the time-frequency cluster showing the higher prior-induced significant modulation in probabilistic settings (~2 - 5 Hz; ~ -500 - 0 ms). The difference between the amplitude values extracted in the random versus mid-validity prior (*Δ theta_mid validity_=theta_random_– theta_mid validity_*) and in the random versus high-validity prior (*Δ theta_high validity_=theta_random_– theta_high validity_*) conditions expresses the degree to which each participant regulates theta pre-stimulus in a different way as a function of prior validity. Since no significant modulation emerged between the mid- and high-validity prior conditions, the two conditions were combined (*Δ theta=mean (Δ theta_mid validity_, Δ theta_high validity_*) to obtain a single*Δ theta*value for use in the subsequent correlation analyses.

### 2.7 Oscillatory amplitude analysis: Left central electrodes

To assess the impact that priors had on motor preparatory activity, we conducted a time-frequency analysis using the signal originating from the electrodes CP1, CP3, CP5, C1, C3, and C5 as a reference for the analyses as they are positioned above the left motor cortex, responsible for signaling decision outcomes in the task as participants always responded with their right hand (Bonnard et al., 2009;Scrivener & Reader, 2022). Then, we performed a frequency per time nonparametric cluster-based permutation tests akin to the one previously described. Furthermore, we computed, for each individual, the mean amplitude value of the time-frequency cluster resulting in being most significantly modulated by the deterministic prior (~12 - 20 Hz; ~ -500 - 0 ms). The difference between the amplitude (*Δ beta_deterministic_=beta_random_– beta_deterministic_*) values obtained in the random versus deterministic condition expresses the degree to which each participant regulates the activity of their left motor area when the prior has random versus deterministic predictive power.

### 2.8 EEG analysis: Brain-to-behavior analysis

To assess whether there was a continuous relationship between the modulations at the behavioral level induced by the expectancy cue and the modulations at the oscillatory level, we conducted several correlation analyses. In all correlation analyses, we corrected the indices extracted from each condition using the random trials as a baseline, as the prior had no predictive power in this condition. To verify the feasibility of the approach, we statistically checked that the random conditions did not differ between empiricists and believers in both fronto-central and motor clusters (Supplementary Figs. S6 and S7). Furthermore, this method aligns with the approach used for time-frequency analyses, in which we evaluated the difference between the various conditions contrasting them with the random condition. Then, we ascertained the presence of a relationship between the extracted starting point regressors and the highlighted midfrontal theta modulation (*Δ theta*) by computing Spearman correlations. A positive value in the starting point regressor highlights a more pronounced shift of the response bias under conditions where the prior gains predictive power. In addition, a negative value in the Δ theta indicates a reduction in theta activity when transitioning from the random condition to those with mid/high validity.

Furthermore, to investigate the spatial distribution of the results, we performed a permutation-based statistic to evaluate which sensor’s activity was able to predict the individual’s predictive tendencies. First, we extracted the*Δ*theta modulation separately for each electrode. Then, we correlated these indices with the starting point regressors. Subsequently, we calculated the number of contiguous electrodes showing a significant relationship and compared it with the number obtained following permutation-based analysis in which we shuffled (n = 1000) the correspondences between*Δ theta*and starting point regressors.

Subsequently, we evaluated whether there was a relationship between the observed modulation in motor areas activity in the deterministic condition and the hit-rate shift in this condition. To this end, we correlated the*Δ beta_deterministic_*value extracted for each participant with the*Δ*hit rate (*Δ*hit rate = hit rate*_random_–*hit rate*_deterministic_*). The rationale is that if the suppression of the beta band moderates the effect of prior-dependent response preparation (i.e., pressing the key associated with target presence), then a relationship with the*Δ*hit rate in the deterministic condition would be expected. Finally, we conducted a data-driven spatial analysis to assess which electrodes’ activity could predict the shift in the hit-rate occurring in the deterministic condition. Specifically, we conducted a Spearman correlation analysis using the*Δ*hit rate as the dependent variable and the*Δ beta*, extracted separately for each electrode, as the predictor. Finally, we calculated the number of contiguous electrodes showing a significant relationship and compared it with the number obtained following permutation-based analysis in which we shuffled (n = 1000) the correspondences between*Δ*hit-rate and the extracted*Δ beta*.

## 3 Results

Human participants (n = 54) performed a simple detection task (Fig. 1A). In each trial, a checkerboard appeared in the lower left visual field, containing (target trials) or not (catch trials) grey circles within its cells. Participants indicated the target presence versus absence via keyboard. The checkerboards were preceded by a symbolic cue indicating the target’s presence probability. There were 4 cue levels indicating different prior predictive power. The mid- and high-validity prior cues indicated, respectively, 63 and 75% target probability, while the random cue could not predict target presence (50%) or absence (50%). Finally, the 100% cue indicated with deterministic precision the certainty of target presence in the upcoming checkerboard presentation.

### 3.1 Prior information modulates the starting point parameter

To assess the impact of probabilistic priors on decision bias, we estimated the Drift Diffusion Model parameters using the HDDM toolbox (Wiecki et al., 2013). Specifically, we fitted a regressor in order to capture the slope of change in the starting point parameter as a function of increased prior validity. Bayesian statistic revealed that the 95% credibility interval derived from the posterior distribution did not overlap zero, indicating the significant impact on the starting point parameter played by prior’s validity (mean starting point regression = 0.058 ± 0.005; q < 0.01;Fig. 1B). This implies that as the prior’s predictive power increased, the starting point was progressively shifted closer to the decision boundary associated with a target-present response. We confirmed this pattern by correlating the starting point regression with three independent measures of decisional bias: RT bias, response bias, and criterion. These measures showed a strong association, demonstrating the parameter’s capacity to capture decisional bias (Supplementary Fig. S4). However, when we analyzed the impact of probabilistic priors on drift rate parameters, we did not find any significant effect. Thus, the increased validity of the prior did not influence the precision nor the rate of evidence accumulation (i.e., drift rate). Crucially, we leveraged the extracted starting point regressors to better characterize inter-individual differences in prior handling within the sample. In this regard, we applied a median split analysis on the starting point regressors to derive the group of participants exhibiting hyper- versus hypo modulation of the starting point based on the provided prior (Fig. 1C). This allowed us to extract the group of*believers*(i.e., participants above the median) and*empiricists*(i.e., participants below the median). Note that all the results obtained were replicated when we divided the participants using a more direct performance index, such as the response bias index (which is calculated considering the different proportions of hits and false alarms across cue levels), further confirming the robustness of the findings (seeSupplementary Materials).

### 3.2 Believers showed a performance advantage in the deterministic condition

To corroborate the goodness of the division performed and investigate the impact played by the two predictive styles in the deterministic conditions, we analyzed raw hit rates and false alarms. First, we computed the hit rate and the false alarm rate (Fig. 1D) separately for trials preceded by the 4 cue types (i.e., random, mid, high, and deterministic).

The repeated-measures ANOVA showed a significant main effect of cue (F_3,__156_= 105.79, p < 0.01) and group (F_1,__52_= 9.63, p < 0.01) as well as a significant interaction between cue and group factors on the hit rate (F_3,__156_= 7.62; p < 0.01). Post-hoc analysis demonstrated that*believers*showed differences in hit-rate across all comparisons (all t_26_> 3.70, all p < 0.001, all Cohen’s d > 0.71, all BF > 33.71). Specifically, they exhibited significantly higher hit rates in the deterministic condition (mean HR_deterministic_= 0.97 ± 0.01), followed by the high-validity condition (mean HR_high__validity_= 0.80 ± 0.02). Finally, the mid-validity condition (mean HR_mid__validity_= 0.69 ± 0.03) showed a higher hit rate compared to the random condition (mean HR_random__validity_= 0.62 ± 0.03). On the contrary, empiricists did not show differences in hit rates across probabilistic conditions (all t_26_< 1.46, all p > 0.15, all Cohen’s d < 0.28, all BF < 0.53). Therefore, the hit rate remained unchanged in the random (mean HR_random__validity_= 0.58 ± 0.04), mid (mean HR_mid__validity_= 0.58 ± 0.04), and high (mean HR_high__validity_= 0.60 ± 0.04) validity conditions. However, they demonstrated a significant increase in hit rates within the deterministic trials (mean HR_deterministic__validity_= 0.81 ± 0.04) compared to the other probabilistic conditions (all t_26_> 7.85, all p < 0.001, all Cohen’s d > 1.32, BF = 56965). Crucially, when comparing performance between empiricists and believers, we found that, while there were no significant divergences in hit rates in the random conditions (t_25_= 0.72, p = 0.47, Cohen’s d = 0.20, BF = 0.34), believers exhibited a markedly higher hit rate percentage compared to empiricists in the mid-validity, in the high validity, and in the deterministic condition (all t_25_> 2.31, all p < 0.03, all Cohen’s d > 0.63, all BF > 2.38). It is noteworthy that the hit rate of believers in the deterministic condition (i.e., 97%) significantly approaches the targeted hit rate level (i.e., 100%). This indicates higher functionality in this specific context for the group that tends to overweigh the prior, which in this specific case correctly weighs the deterministic prior (100% target occurrence).

The analysis conducted on false alarms highlighted a significant main effect of cue (F_2,__104_= 40.51, p > 0.01) and group (F_1,__52_= 4.66, p = 0.04) as well as a significant interaction of prior by group factors (F_2,__104_= 7.19, p < 0.001). Post-hoc analyses revealed that believers had a false alarm rate that increased with the predictive power of the prior (all t_26_> 2.49, all p < 0.02, all Cohen’s d > 0.48, all BF > 2.7). Indeed, believers exhibited higher false alarms in the high probability condition (FA high validity = 0.35 ± 0.04), followed by the medium condition (FA mid-validity = 0.20 ± 0.03), and finally the random condition (FA random validity = 0.16 ± 0.03). Conversely, empiricists showed an increase in false alarms in the high validity prior condition (FA high validity = 0.20 ± 0.03) compared to the mid- (FA mid validity = 0.13 ± 0.02) and random- (FA random validity = 0.14 ± 0.02) conditions (all t_26_> 5.01, all p < 0.01, all Cohen’s d > 0.96, all BF > 722), which did not differ from each other (t_26_= -1.18, p = 0.25, Cohen’s d = -0.25, BF = 0.38). Comparing empiricists and believers, it emerged that the false alarm rate is higher in believers in the high probability condition (t_25_= 2.75, p = 0.008, Cohen’s d = 0.75, BF = 5.57), while not differing in the other two conditions (all t_52_< 1.81, all p > 0.07, all Cohen’s d < 0.49, all BF < 1.05).

Overall, these results demonstrated that believers and empiricists did not show differences in their baseline perceptual performance as both hit and false alarm rate were not different in the random condition. However, when prior information gains predictive power, believers exhibited different modulation rates, resulting in higher hit rates and more false alarms. This increased reliance on prior information indicates a tendency among believers to adopt a more liberal strategy. Consequently, they were more likely to report the presence of the target, which leads to both higher hit rates in target-present trials and an increased number of false alarms in target-absent trials. Crucially, this behavioral modulation was present regardless of the time period considered, testifying to a differentiation in the predictive strategies that was evident from the very beginning of task execution (seeSupplementary Materials). Overall, these results align with the modulation of the starting point (but not the drift rate) observed using Drift Diffusion analysis. Crucially, the different weight assigned to prior played a significant role in perceptual performance in the deterministic condition. Here, heavily weighting the prior becomes crucial, given its 100% predictive validity; therefore, basing responses on it becomes functional. Importantly, empiricists, who tend to weigh the prior less, exhibit a hit rate far from the 100% target (i.e., 81%). In contrast, believers approach this value closely (i.e., 97%), indicating that they have appropriately weighted the prior in this specific context.

### 3.3 Believers exhibited faster prior-congruent responses

We have also ascertained whether RTs could be modulated in the two extracted groups (Supplementary Fig. S5). The rationale is that the RTs for prior congruent choice (i.e., response present in the high probability condition) should be faster compared to prior incongruent choice (i.e., response absent in the high probability condition). Concerning present responses, the ANOVA showed a null effect of the group factor (F_1,__52_= 2.87, p = 0.10) while both cue (F_3,__156_= 85.92, p < 0.01) and cue × group interaction (F_3,__156_= 4.70, p < 0.01) were significant. Post-hoc tests revealed that Empiricists responded faster in the deterministic condition (RT deterministic = 0.54 ± 0.04, all t_26_> 4.85, all p < 0.01, all Cohen’s d > 0.93, all BF > 503) relative to all other conditions (RT random = 0.72 ± 0.05; RT mid = 0.71 ± 0.04; RT high = 0.69 ± 0.05) and were also quicker in the high-predictive condition compared to the mid-predictive condition (t_26_= 2.48, p = 0.02, Cohen’s d = 0.48, BF = 2.64). Believers, on the other hand, showed significant differences across all conditions, reflecting a gradual acceleration in RTs as cue predictability increased (RT random = 0.69 ± 0.04; RT mid = 0.64 ± 0.04; RT high = 0.58 ± 0.03; RT deterministic = 0.39 ± 0.03; all t_26_> 3.34, all p < 0.01, all Cohen’s d > 0.64, all BF > 15). When comparing the two groups, Believers responded faster in the high-predictive condition (t_52_= 1.97, p = 0.05, Cohen’s d = -0.53, BF = 1.37) and in the deterministic condition (t_52_= 2.57, p = 0.01, Cohen’s d = -0.70, BF = 3.85), but not in the mid-probability condition (t_52_= 0.53, p = 0.59, Cohen’s d = -0.14, BF = 0.55). These results closely mirror the pattern observed for hit rates, as in the high-predictive and deterministic conditions, Believers demonstrated an advantage in leveraging prior information, allowing them to respond more quickly when the stimulus was present.

Regarding absent responses, the ANOVA showed a significant cue effect (F_2,__104_= 23.79, p < 0.01), while the group factor was not significant (F_1,__52_= 0.04, p = 0.98). Crucially, we found interaction between cue predictability and group (F_2,__104_= 4.38, p = 0.01). Post-hoc tests showed that Empiricists had slower RTs in the high-predictive condition compared to the random condition (RT random = 0.68 ± 0.03; RT high = 0.71 ± 0.04, t_26_= 2.84, p < 0.01, Cohen’s d = 0.55, BF = 5.26), while all other comparisons were not significant (all t_26_< 1.44, all p > 0.13, all Cohen’s d < 0.29, all BF < 0.58). In contrast, Believers exhibited a progressive slowing of RTs as cue predictability increased, with significant differences across all comparisons (RT random = 0.68 ± 0.03; RT mid = 0.70 ± 0.03; RT high = 0.71 ± 0.04; all t_26_> -3.61, all p < 0.01, all Cohen’s d > 0.69, all BF > 28). This pattern aligns with the expected task dynamics, where absent responses conflict with the predictive information provided by the cue, leading to longer decision times. All in all, these analyses demonstrated that even the analysis of RT demonstrated a strong prior-driven factor, with a speeding up of prior-congruent response. This effect was, in turn, modulated by the group factor, as the prior congruency effect was stronger in Believers compared to Empiricists.

### 3.4 Pre-stimulus theta oscillations shape response bias in probabilistic conditions

**Fig. 2.**
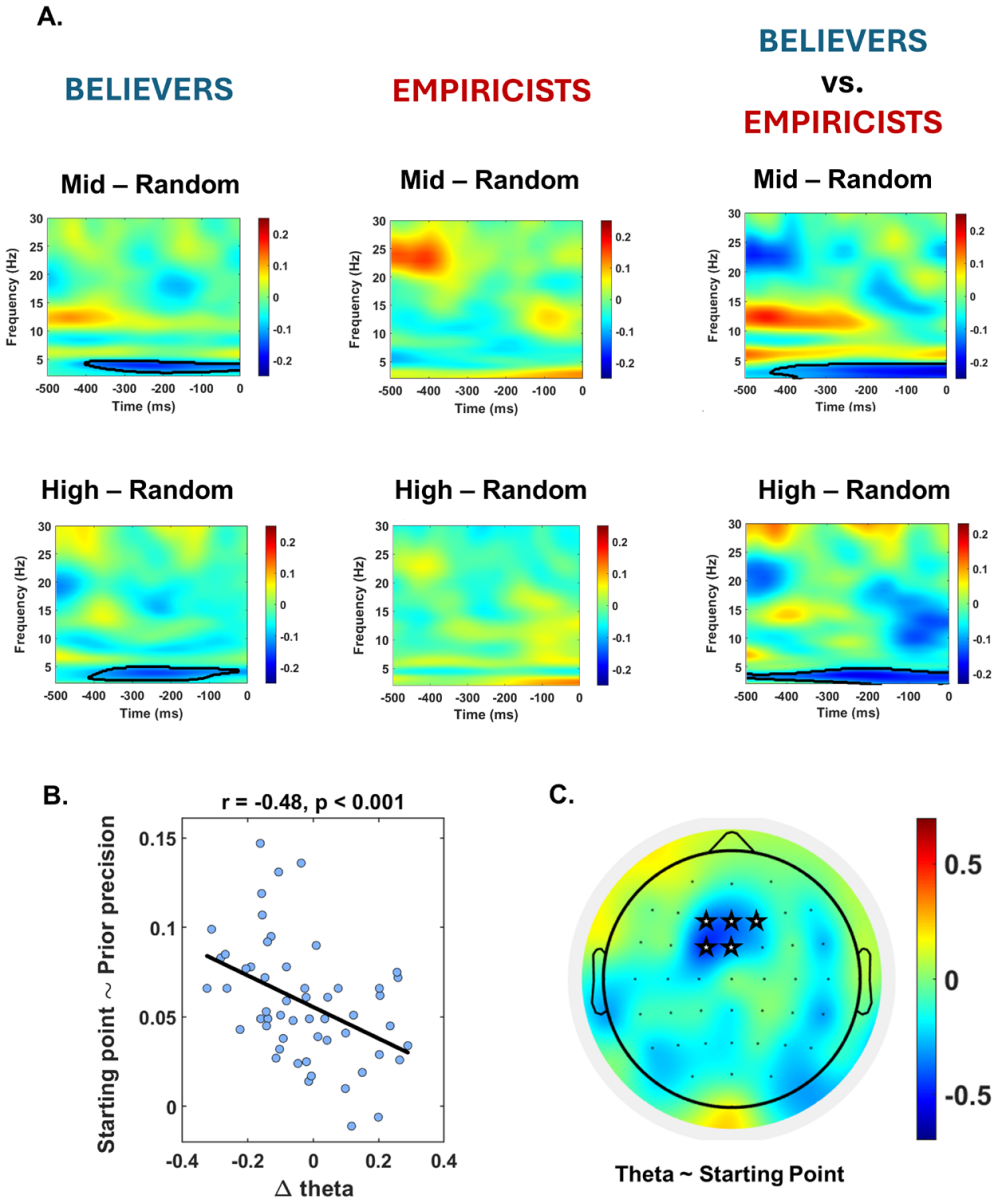
Association between behavioral and neural markers of prior information in the probabilistic condition. Time-frequency map of the pre-stimulus (−500, 0 ms) amplitude difference between mid/high versus random validity registered in mid-frontal regions. Time 0 refers to stimulus onset. Black contours denote cluster resulting significant from statistical analysis. (A) It is noticeable that there is a lower activation in pre-stimulus theta amplitude in conditions where prior information indicates the probability of target presence compared to random conditions. Importantly, this effect is discernible exclusively within the participant group (believers) characterized by a heightened reliance on prior information in decision-making. (B) Correlation between Δ theta amplitude and the individual estimate of the starting point regression. A significant negative correlation is observed between individual differences in prestimulus theta amplitude during mid/high and random prior validity trials (Δ theta amplitude) and the starting point shift. This association indicates that the lower the activation of theta oscillations when the prior acquires predictive power, the more pronounced the subsequent shift in starting point. (C) Spatial analysis reveals that oscillatory activity located in the theta band, originating in midfrontal electrodes, is able to predict prior-driven behavioral changes. Theta activity in the rest of the brain was found to be unrelated to this effect. The colorbar represents the correlation coefficient.

We assessed whether preparatory activity in the fronto-central regions was modulated by prior information by running a frequency per time non-parametric permutation test across the pre-stimulus window on the amplitude difference between 1) the random and the mid-validity prior condition, 2) the random and the high-validity prior condition, and 3) the random and the deterministic prior condition. The analysis revealed a significant effect in the pre-stimulus period. First, a lower activation in the 2–5 Hz band oscillation was found for probabilistic (both 63% and 75%) relative to the random (i.e., 50%) condition. Crucially, this effect was present only in the*believers’*group (p_cluster__mid vs. random_= 0.04, time period -400–0 ms; p_cluster high vs. random_= 0.025, time period -400–20 ms), while no significant modulation was found in the*empiricists*group (Fig. 2A) or when considering the whole sample (Supplementary Fig. S8). Interestingly, there were no significant differences between the mid- and high-validity conditions in any of the groups considered. Secondly, we found a difference between the random and the deterministic condition in the beta frequency range for both empiricists and believers, as well as across the entire sample (Supplementary Fig. S9), while no effect was found in the theta band in this comparison.

Next, we analyzed whether this neural effect could explain the different weight assigned to prior across our sample in the probabilistic conditions. If the lower amplitudes of theta rhythms following the probabilistic, relative to the random trials, found in the believers’ group are determined by a higher weight assigned to prior and reduced monitoring of sensory input, it is reasonable to expect an association between theta amplitude modulation and response bias. Spearman’s correlation analyses conducted considering the whole sample showed a significant correlation between the degree of individual starting point shift and the extent of individual theta amplitude modulation [Spearman (Starting Point ~ Δ theta amplitude) = -0.48, p < 0.01, BF = 28.44;Fig. 2B]. Furthermore, data-driven analysis highlighted the spatial specificity of the effect, demonstrating that theta oscillations originating from fronto-central areas show an association with the modulation of the criterion, whereas this association does not hold in other cortical regions (Fig. 2C). Thus, these results suggest that midfrontal theta amplitude regulation is a crucial marker of response strategy tuning when prior information conveys probabilistic, rather than deterministic, information.

### 3.5 Deterministic prior exerts its behavioral effect by modulating beta oscillations in motor regions

**Fig. 3.**
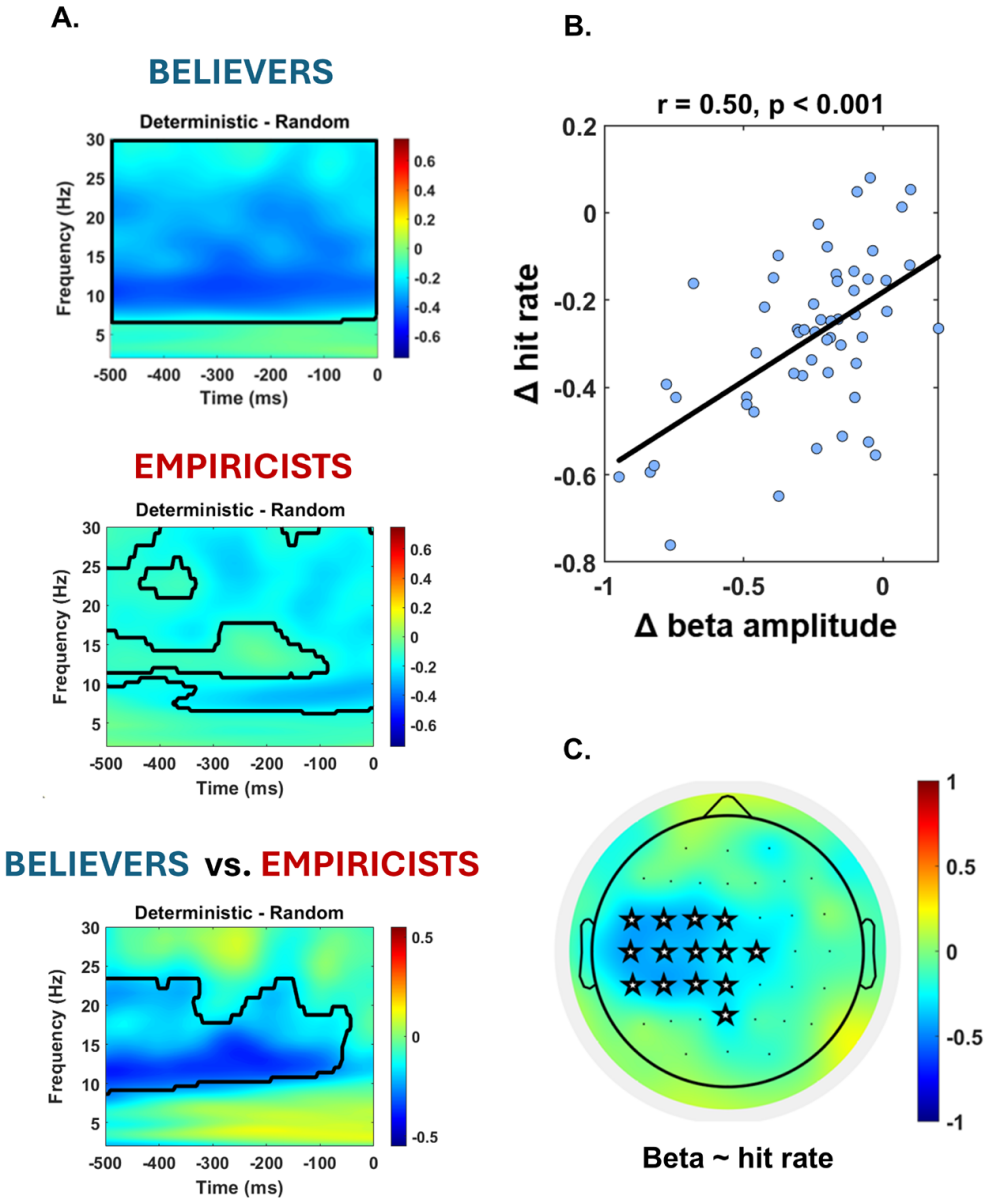
Association between behavioral and neural markers of prior information in the deterministic condition. (A) Time-frequency analyses revealed strong desynchronization in the alpha-beta band under deterministic conditions relative to conditions where the prior had random predictive power. This effect was present in both the believers and empiricists groups. However, contrasting the activation map of believers and empiricists, we identified a significantly greater effect in believers that over-desynchronize beta activity in the prestimulus time. (B) The observed desynchronization was able to predict the behavioral change between the deterministic and random condition. Specifically, the greater the desynchronization in the beta band, the higher the increase in hit rate in deterministic versus random condition. (C) Spatial analysis reveals that oscillatory activity located in the beta band, originating in the left central electrodes, is able to predict prior-driven behavioral changes. Beta activity in the rest of the brain was found to be unrelated to this effect. The colorbar represents the correlation coefficient.

We assessed whether preparatory activity in the left motor cortex undergoes changes based on prior induction in order to prepare the right-hand motor response. To this end, we evaluated, using a time-frequency analysis, the presence of a differential pattern of activity in 1) the random and the mid-validity prior condition, 2) the random and the high-validity prior condition, and 3) the random and the deterministic prior condition. First, we did not observe any significant modulation in the motor areas in probabilistic contexts, neither when considering the total sample nor when dividing the groups based on the predictive strategy (Supplementary Fig. S10). Instead, the non-parametric permutation test revealed a consistent suppression across a broad spectrum of frequencies encompassing the alpha-beta band in the deterministic conditions. Crucially, this suppression was present in both the*empiricists*and*believers’*group (Fig. 3A, p clusters < 0.001, time period = -500–0 ms), as well as in the overall sample (Supplementary Fig. S11). Nevertheless, upon contrasting the activation maps in the two groups, we observed that believers exhibited a more pronounced beta band desynchronization during the prestimulus period (Fig. 3A, p cluster < 0.01, time period -500–50 ms). In order to investigate whether the beta shift highlighted at the oscillatory level coded for the increase in hit-rate highlighted in the deterministic condition, we ran a correlation analysis that demonstrated that the degree of the beta suppression significantly correlates with hit rate shift (r = 0.50, p < 0.01, BF = 2238;Fig. 3B). In particular, participants displaying greater beta desynchronization in deterministic trials are those who outperform in this condition. The spatial analysis conducted reinforces the hypothesis that this effect originates from a motor preparation effect (Fig. 3C). In particular, the beta desynchronization originating from the cluster that includes left central electrodes (i.e., motor regions) emerged as the predictor of the observed behavioral differences in the deterministic condition. Conversely, electrodes located elsewhere did not capture these behavioral changes. Furthermore, this beta suppression also correlates with the facilitation in reaction times observed in the deterministic condition compared to the random one (Supplementary Fig. S12).

All in all, the results revealed a substantial dissociation between the neuro-behavioral markers associated with probabilistic and deterministic settings. In probabilistic scenarios (mid and high prior validity), the theta rhythm originating from fronto-central regions influenced the impact of prior information on perceptual decision-making. On the contrary, in deterministic situations, there is an involvement of the motor system, exhibiting a robust desynchronization across the beta band. Crucially, this desynchronization predicts the observed shift in behavioral performance. Finally, these preparatory markers were susceptible to interindividual differences in prior processing, as they clearly emerged only when considering the*believers*rather than the*empiricists*group.

## 4 Discussion

To investigate the behavioral and neural underpinnings of predictive processing, we recorded the EEG activity of human observers while engaged in a probabilistic detection task. In this task, we manipulated the validity of prior information across random (50%), mid-probability (63%), high-probability (75%), and deterministic (100%) levels. Drift Diffusion Model analysis suggests that participants successfully incorporated expectation-like information into their decision-making process. In particular, the manipulation of perceptual expectations in the probabilistic conditions had a strong impact on response bias, influencing the starting point (but not the drift rate) of the evidence accumulation.

Moreover, we examined the influence of the deterministic prior, predicting the target with 100% certainty, on behavioral performance compared to the probabilistic priors used in the other conditions. We observed an improvement in performance, specifically in terms of hit rate, in the deterministic compared to the probabilistic trials. This result can be interpreted in light of the significantly simpler nature of this condition compared to the others, given that, independently of the ability to correctly discern the sensory input, the prior provided certainty on the validity of the response. In this scenario, the optimal strategy involved placing “blind” full trust in the prior information and simply reporting the presence of the target. Notably, in this condition, the need to test the prior against sensory evidence was disadvantageous, as the precision of the sensory signal (titrated to 70% perceptual accuracy) was very far from the prior precision (100%).

Furthermore, perception was not only influenced by the objective validity of the priors but also by the subjective evaluation assigned to their predictive power. Numerous studies (Powers et al., 2017;Schmack et al., 2013;Stuke et al., 2021;Tulver et al., 2019) have highlighted substantial interindividual variations within the general population concerning the weighting of prior information. According to a novel bio-behavioral model (Tarasi, Trajkovic, et al., 2022), individuals exhibit a spectrum of predictive styles. Decision-makers at one end of this spectrum tend to attribute higher precision to prior information, leading to a greater propensity for utilizing it. In contrast, observers at the opposite end tend to adopt an empirical, bottom-up strategy that reduces the influence of prior information in favor of bottom-up inputs (Tarasi et al., 2021;Ursino et al., 2022). Building upon this context, we used the output from the fitted Drift Diffusion Model to extract the subgroup of individuals who exhibited a more pronounced modulation of decisional bias in response to prior information (i.e.,*believers*) compared to those with a lesser modulation (i.e.,*empiricists*).

Corroborating analyses of raw hit and false alarm rate confirm distinct behavioral performance between the two identified groups. Believers, who showed a greater modulation of the starting point parameter, exhibited increased hit rates and false alarms corresponding to the predictive power of the priors. This pattern suggests a bias induction, as believers more frequently reported the presence of the target in both target-present and target-absent trials.

In contrast, empiricists demonstrated less modulation based on prior information, indicating a lower weight given to the prior. Crucially, in analyzing the deterministic condition, we found that the*believer*group demonstrated comparatively improved performance, highlighted by a higher hit rate, when the prior consistently predicted the target’s appearance, relative to the*empiricist*group. This improvement indicates that the believers, who are more inclined to weigh prior information, effectively assigned higher value to the deterministic prior (100% target occurrence), thereby enhancing their performance in this scenario. In contrast, empiricists appear to treat the deterministic cue similarly to a probabilistic one, as reflected in their substantially lower accuracy (i.e., 81%) compared to the expected 100%. A similar pattern emerged when examining reaction times. Believers consistently showed greater facilitation as the predictive power of the priors increased, culminating in very low reaction times in the deterministic condition, where they once again outperformed empiricists.

Importantly, we demonstrated that the oscillatory fingerprints characterizing probabilistic contexts (i.e., where prior conveys a probability of target presence, such as in the mid and high prior validity conditions) and the deterministic context (i.e., when prior conveys a certainty about target presence) were different. In probabilistic contexts, theta modulation marked prior processing. Specifically, reduced theta amplitude was observed in conditions in which priors had mid/high prior predictive power compared to random conditions, suggesting a reduced monitoring impact and an increased reliance on prior information. Importantly, this pattern was exclusive to the*believers*group. As*believers*transitioned from random to mid/high validity prior conditions, a pronounced lack of activation of theta oscillation was evident. In contrast, within the*empiricists*group, the level of theta oscillations remained unaltered despite the increase in prior predictive power. The lack of engagement of theta activity in the*believers*as the prior becomes more precise points toward a diminishing monitoring on the perceptual process, leading it to become more prior-driven and less constrained by incoming stimulation. This result aligns with earlier findings that illustrated how individuals manifesting lower frontal theta power were identified as more susceptible to bias in a learning task (Cavanagh et al., 2013). Interestingly, theta rhythms remained consistent between the mid- and high-validity contexts. This highlights a challenge for human observers in fine-tuning the observed theta-based mechanism, possibly stemming from a suboptimal alignment between objective and subjective prior precision (Rahnev & Denison, 2018). Another noteworthy aspect to consider is that the*empiricist*group maintained a consistent level of theta activity across different conditions. It is possible to hypothesize that, instead of enhancing the level of monitoring as the prior validity increases, individuals with a cognitive style inclined toward underweighting priors might choose to just downplay their influence, maintaining the same level of monitoring regardless to the provided prior. Similarly, we found a relationship with reaction times, where believers were faster to respond when providing a congruent response with the prior information received.

To further validate the involvement of theta oscillations in the modulation of decision-making strategy, we conducted correlation analyses to establish a link between this neural marker and behavioral findings. The results robustly demonstrate that the extent of theta reduction correlates significantly with the starting point modulation. Furthermore, theta modulation was associated with RT bias, specifically the speeding up versus slowing down of RTs following a congruent (e.g., present) versus incongruent (e.g., absent) response relative to the probabilistic prior received (e.g., high probability). This finding is consistent with recent studies linking reaction times and theta activity (Beldzik & Ullsperger, 2024).Kaiser et al. (2022,^2023^) reported a negative relationship between theta activity and RTs. In contrast, we found that reduced theta activation was associated with faster RTs. This apparent contradiction may stem from the different paradigms used. In theKaiser et al. (2022)study, participants performed a cued change-signal task, where frontal theta power increased selectively during successful conflict resolution, resulting in faster response times. In our task, a reduction in theta oscillations was observed in the group using a prior-driven approach, likely reducing proactive monitoring and control, which in this context facilitates faster responses when the prior is predictive in a probabilistic setting. Additionally, the data-driven spatial analysis conducted revealed that this association was observed exclusively for theta activity recorded from fronto-central electrodes. In contrast, electrodes located in other spatial positions showed no significant effect in explaining the change in predictive strategies. The spatial specificity of these effects lends support to the hypothesis that the observed theta activity is indicative of the extent of cognitive monitoring (dis)engagement that participants employ during the integration of probabilistic priors into perceptual decision-making. This is suggested by the involvement of theta originating from the same region in cognitive control, response inhibition, and bias regulation (Cavanagh et al., 2013;Cohen & Donner, 2013;Cooper et al., 2019;Duprez et al., 2020). It is worth noting that the effect manifests in low theta frequencies and also spans the range traditionally associated with delta. This is in line with studies showing that low theta is linked to enhanced executive functions, control, and guidance of the perceptual process (Bender et al., 2019;Helfrich et al., 2017;Wolinski et al., 2018).

Fronto-central theta oscillations did not impact decision-making strategies in the deterministic condition. This contextual shift would change the mechanisms underlying the use of the prior. In probabilistic conditions, the reduced monitoring is linked to reliance on the prior, while in deterministic conditions, more automatic mechanisms tied to motor preparation come into play, as participants can simply pre-program the response to be provided (i.e., pressing the button associated with stimulus presence reporting). Indeed, by comparing the activation patterns of the deterministic and the random condition in the left motor areas, we identified a specific neural pattern that differentiated them. Specifically, we observed a strong desynchronization peaking in the beta band. Additionally, the extent of this desynchronization was able to predict the improvement in hit rate observed in the deterministic compared to the random condition. This result aligns with previous literature demonstrating that beta activity can predict participants’ choices (Donner et al., 2009) and that beta power increases are observed in the contralateral motor cortex during movement inhibition and motor-slowing tasks (Muralidharan et al., 2019). These increases have been linked to heightened decisional thresholds, supporting the notion that beta oscillations play a critical role in motor control and decision-making processes (Kaiser & Schütz-Bosbach, 2019). Consistent with these findings, our study reveals a strong relationship between the extent of beta power decrease and movement facilitation in our task. Furthermore, the spatial analysis carried out supports the idea that this effect stems from a motor preparation effect. Specifically, the beta desynchronization deriving from the cluster containing left central electrodes (i.e., motor regions) emerged as the only predictor of the observed behavioral differences in the deterministic condition. Crucially, this neural effect, although present in both groups, was more consistent in the*believers’*group. This diversification in the pattern of cortical activation explains the mechanism that leads*believers*to achieve better performance in the deterministic context compared to the*empiricist*group. This result underscores that the appropriateness of a predictive style is strictly context-dependent. Specifically, a deterministic scenario favors individuals inclined to adopt a prior-driven style, enabling them to optimize the stringent rules within that specific context. Moreover, a key factor contributing to the success of this strategy is that, within the implemented experimental paradigm, the predictive information conveyed by priors was accurate. In follow-up studies, it would be intriguing to investigate whether in contexts where priors convey deterministic, but false, information, there is a reversal of the effect. One might expect that in such scenarios,*empiricists*could outperform*believers*by achieving better performance. It is noteworthy that this differentiation in beta oscillations was observed only in the deterministic condition, but not in the probabilistic conditions. Since both responses were made with the right hand, motor preparation for the two fingers is presumably overlapping in probabilistic scenario. However, it should be noted that we contrasted the time-frequency activity against the neutral condition (i.e., random), where no action preparation is possible due to the equiprobability between the two action plans. In contrast, the probabilistic conditions would, in principle, allow for the preparation of the action congruent with the prior (e.g., preparing to press “K”). While this preparatory mechanism could still be present, it does not appear sufficient to manifest as a significant effect in our data. Follow-up studies could employ a paradigm where the two alternative responses are mapped to left- and right-hand movements to better outline the lateralization due to probabilistic prior (Kelly et al., 2021).

Furthermore, this finding aids in interpreting the lack of fronto-central theta differentiation within the deterministic condition between the two groups, which appears to be driven by distinct mechanisms. For empiricists, it reflects their consistent disregard for priors, regardless of predictive power, and a reliance on sensory input, leading to stable theta activity across conditions. For believers, the absence of theta modulation in the deterministic condition reflects their ability to appropriately weigh the context, switching to a more efficient response strategy when priors are fully reliable, likely tied to motor anticipation.

Moreover, we demonstrated that these prior-dependent modulations were evident from the beginning of the task and remained stable throughout the experiment. This finding enhances our understanding of the mechanism at play, suggesting that the identified predictive strategies (*believers vs. empiricists*) are not time-dependent. In other words, individuals do not gradually shift toward one strategy as the experiment progresses. Instead, these indices seem to reflect intrinsic predispositions that drive participants toward either overweighting or underweighting prior-related information. This result aligns with recent research (Tarasi et al., 2023), which showed that the degree of autistic and schizotypic traits can predict the adoption of an empiricist or believer strategy, respectively. These findings support the notion that individual predispositions underlie the initial preference for one strategy over another.

Finally, we demonstrated that posterior areas did not exhibit differentiation in activity related to the provided prior, except for a general desynchronization in alpha oscillations observed in the believers group. In contrast, posterior theta oscillations showed no effect, supporting the notion that theta-related mechanisms reflect higher-order cognitive processes localized to areas typically associated with cognitive control, rather than extending to visual regions. Future studies should explore whether posterior areas become involved in processing the prior at later stages, during stimulus processing, under the employed protocol.

All in all, we have demonstrated that deterministic contexts are marked by a desynchronization mechanism in the beta band originating from the left motor area controlling motor hand response. This process likely signifies preparatory activity occurring in the motor area, preprogramming the action associated with the deterministic prior (i.e., reporting target presence). To corroborate this interpretation, this suppression is stronger in*believers*and the level of beta desynchronization correlated with the behavioral benefit observed in the deterministic condition. On the contrary, theta oscillations came into play in probabilistic settings. Theta oscillations are implicated in cognitive control, acting as a regulatory mechanism that influences the selection and implementation of strategies during decision-making processes (Cohen & Donner, 2013;Ertl et al., 2013;Rajan et al., 2019;Riddle et al., 2020;Senoussi et al., 2022;Wendiggensen et al., 2022). Specifically, theta oscillations would contribute to the oversight of response tendencies, ensuring a dynamic interplay between incoming sensory information and prior knowledge. In this context, theta oscillations could serve as a signal monitoring system, modulating the influence of prior knowledge on decision-making. The significantly reduced midfrontal theta activation in the group that heavily relies on priors suggests a decrease in signal monitoring process, which would become more prior-driven and less constrained by incoming stimulation. Follow-up studies will investigate whether this involvement encompasses a nuanced control mechanism that navigates the balance between exploiting existing knowledge and adapting to the new perceptual demands, ultimately contributing to the flexibility and adaptability of perceptual decision-making. Additionally, the correlational nature of the presented evidence highlights the necessity of introducing causal approaches to ascertain whether theta desynchronization precedes an over-evaluation of decisional priors (Bertaccini et al., 2023;Di Luzio et al., 2022;Tarasi, Turrini, et al., 2024). This could be achieved through noninvasive neurostimulation inhibitory protocols [e.g., continuous theta burst stimulation (Wischnewski & Schutter, 2015)] applied to brain regions where the theta effect manifested (i.e., fronto-central areas) to evaluate whether the stimulation results in a liberalization in the use of priors. Finally, the exacerbation of this prior-driven theta unleashing could potentially account for psychopathological disorders. Notably, theta oscillatory dynamics exhibit disruptions in various clinical populations when compared to the performance of healthy adults, representing a potential index of cognitive vulnerabilities across different disorders (McLoughlin et al., 2022). In particular, positive symptoms observed in schizophrenia could be interpreted as a deficit in monitoring predictive information. For instance, individuals with schizophrenia are characterized by an overweighting of prior knowledge (Corlett et al., 2019;Kafadar et al., 2022;Powers et al., 2017;Tarasi, Trajkovic, et al., 2022) and demonstrated impairments in performance on tasks related to reality monitoring, with these deficits being concomitant with specific reductions in neural activity in the medial prefrontal cortex (Garrison et al., 2017). Crucially, theta activity is selectively disrupted in schizophrenic patients during proactive cognitive control trials (Ryman et al., 2018). It would be intriguing to assess whether providing a probabilistic prior leads individuals with schizophrenia to exhibit an extreme lack of engagement of the signal monitoring system, resulting in reduced theta activity. This investigation could shed light on the relationship between altered predictive processing, as reflected in theta oscillations, and the manifestation of clinical symptoms in psychiatric populations.

## Supplementary Material

Supplementary Material

## Data Availability

The data and code supporting the findings of this study will be made available upon reasonable request to the corresponding author.
