# Supplementary material for: Oscillatory signatures of monitoring and anticipatory strategies for probabilistic vs deterministic cues
Source: Imaging Neurosci (Camb). 2025 Mar 7;3:imag_a_00496. doi: 10.1162/imag_a_00496 (PMC12319955; doi:10.1162/imag_a_00496)
Supplement: Supplementary Material [file imag_a_00496-supp.pdf]

SUPPLEMENTARY MATERIALS

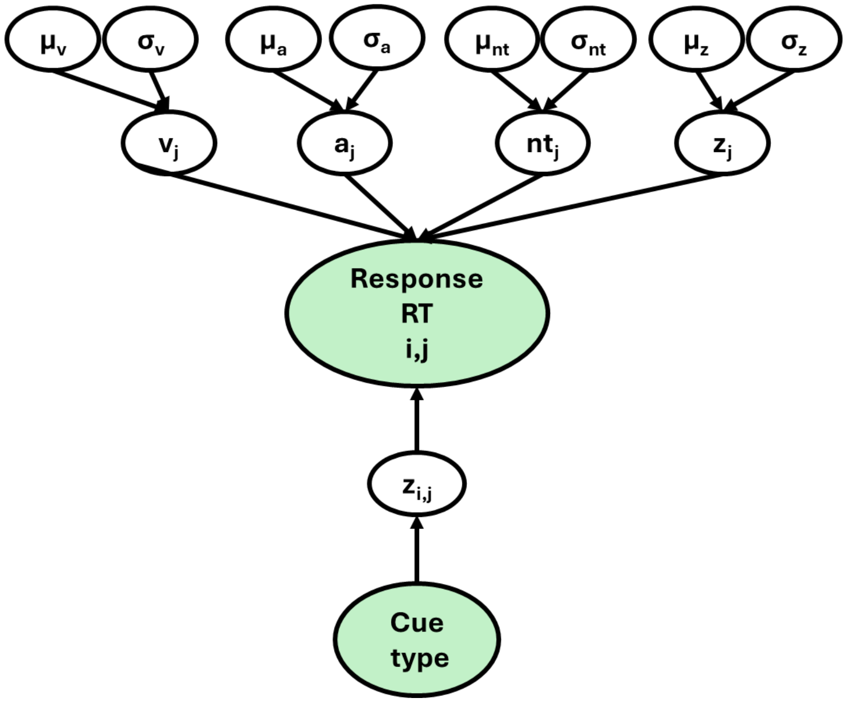

Figure S1 A graphical representation of the HDDM with trial-by-trial regressors.

Decision parameters such as drift rate (v), decision threshold (a), non-decision time (nt), and starting point (z) were estimated for both the group (group mean μ and variance σ) and individual subjects (j). Green nodes indicate observed data, which includes trial-specific behavioral data (accuracy, RT) and cue type (Random, Mid, High probability). The trial-by-trial variations of z were influenced by the cue type.

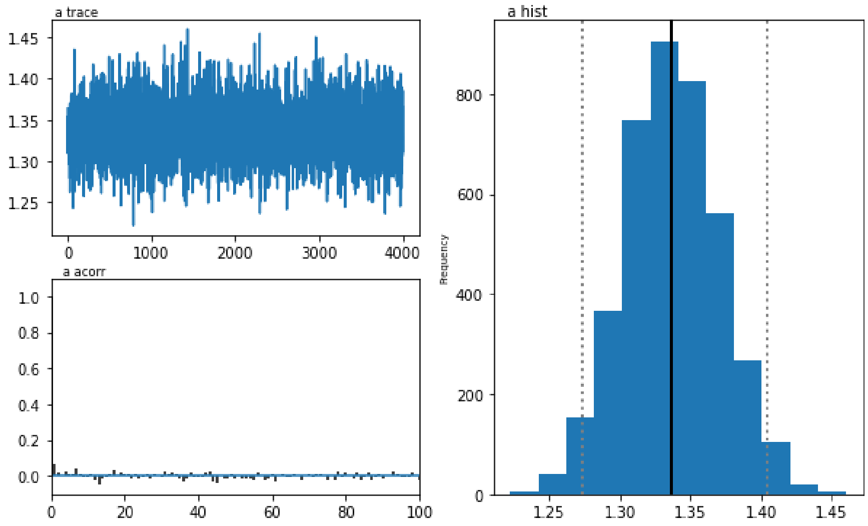

Decision Boundary

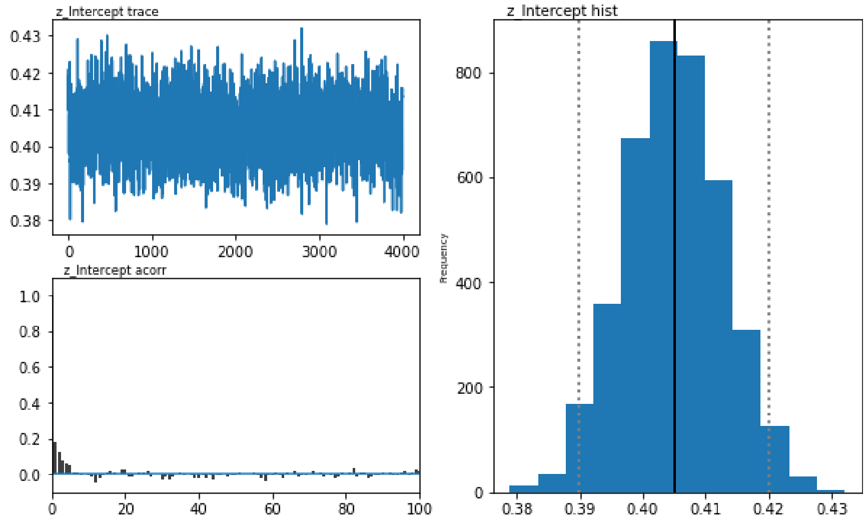

Starting Point Intercept

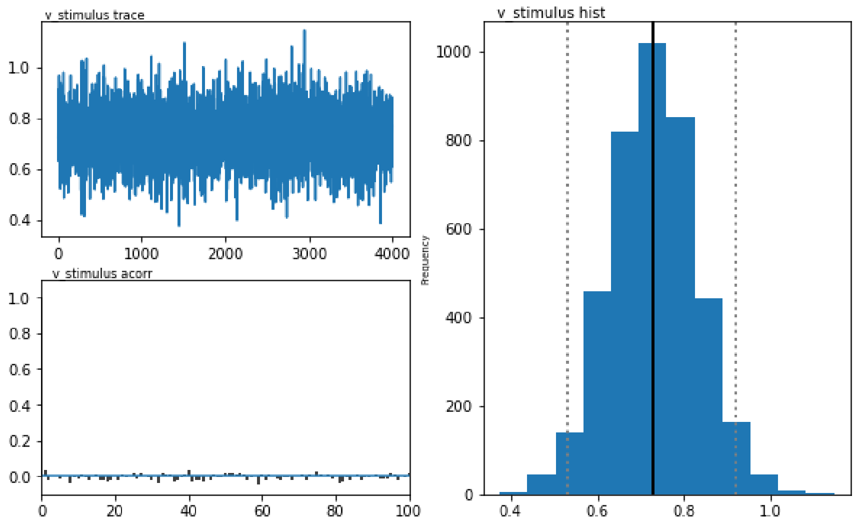

Drift Rate

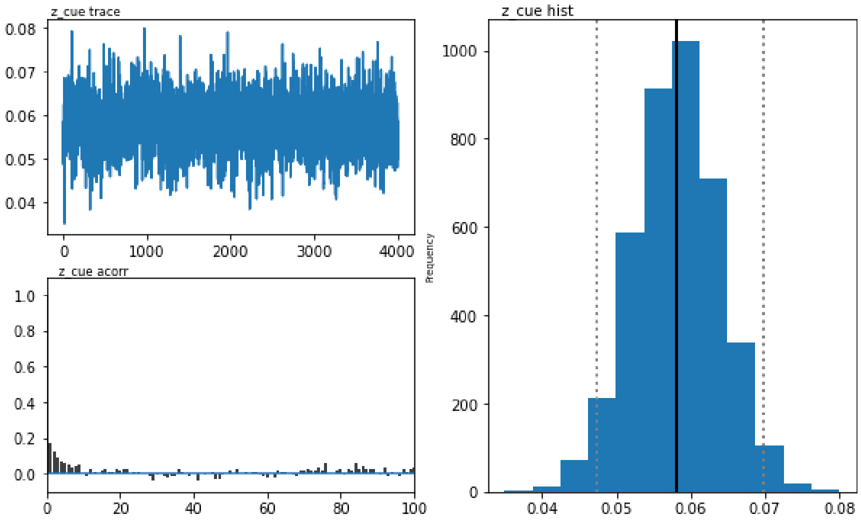

Starting Point Regressor

Figure S2. Trace, Autocorrelation, and Posterior Distribution
The figure displays the traces, autocorrelations, and posterior distributions of the extracted DDM parameters. These diagnostics assess the reliability of parameter estimation. The chains are well-mixed, showing no sudden drifts or abnormal patterns. Autocorrelation is consistently low, and the posterior distributions are well-defined, following a normal-like shape. Together, these observations indicate a robust reconstruction of the parameters.

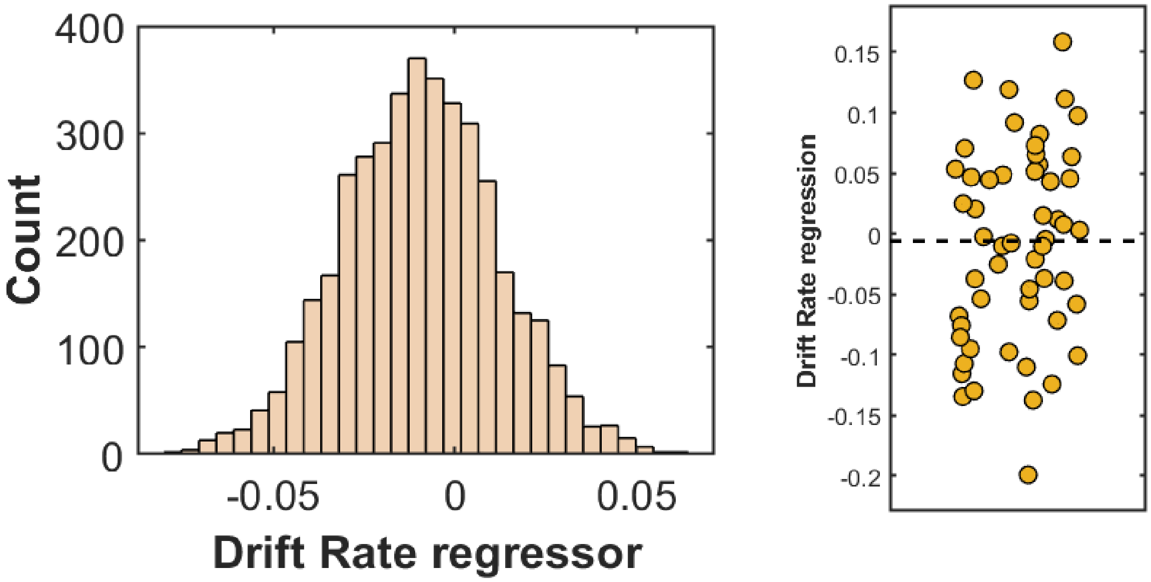

Figure S3. Prior Information Does Not Influence the Drift Rate Parameter

On the left, we depicted the posterior distribution of the drift rate regression parameter. The drift rate parameter was not modulated by the validity of the prior (q = 0.41). By extracting the credibility intervals, we showed that the coefficient of the drift rate regression overlaps with zero. This suggests that, as the validity of the prior regarding target presence increases, there is no corresponding modulation in the capacity to accumulate information in a faster or more precise way.

On the right, we represented the mean of the posterior distributions for each participant for the drift rate regressor parameter. While there are significant interindividual differences in the degree of this parameter, it is evident that the distribution of values is centered around zero, further confirming the absence of any prior-related effect on drift rate.

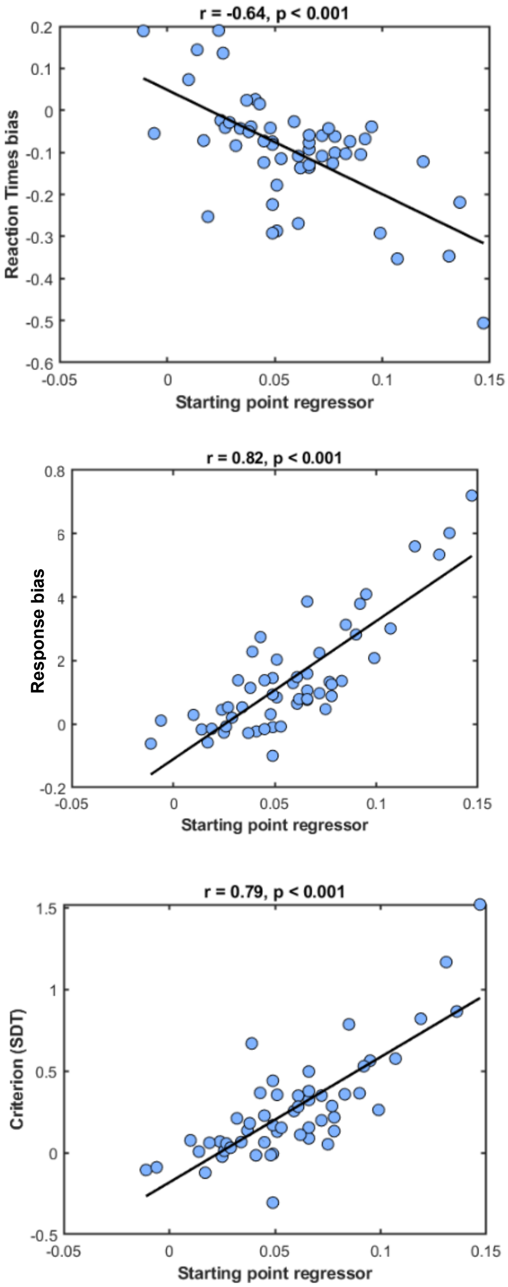

Figure S4 Correlation Between Starting Point Regressor and Decision Bias Metrics

The figures illustrate correlations between the starting point regressor and other metrics, including “rawer” measures of perceptual performance and outputs from independent computational models applied to perception. These analyses demonstrate how the starting point regressor effectively captures decision bias patterns in a single composite measure.

First, we found a robust relationship between the regressor and RT bias. This supports the presence of a facilitation effect in RTs (as shown in the main text), where participants responded faster when their responses were congruent with the prior. The magnitude of this RT bias strongly correlates with the starting point regressor.

Additionally, the starting point regressor is linked to changes in hit rates as well as false alarm as the predictive power of the prior increases. Specifically, larger differences in hit rates and false alarm are reflected in higher positive values of the regressor.

To further validate these findings, we used an independent approach: Signal Detection Theory (SDT), a widely adopted framework in perceptual decision-making. From this model, we extracted the criterion index, which synthesizes information about decision bias (Tarasi, di Pellegrino, et al., 2022)*. Importantly, we observed a strong association between the criterion index and the starting point regressor. This underscores the regressor's robustness and precision in capturing decision bias within the task.*

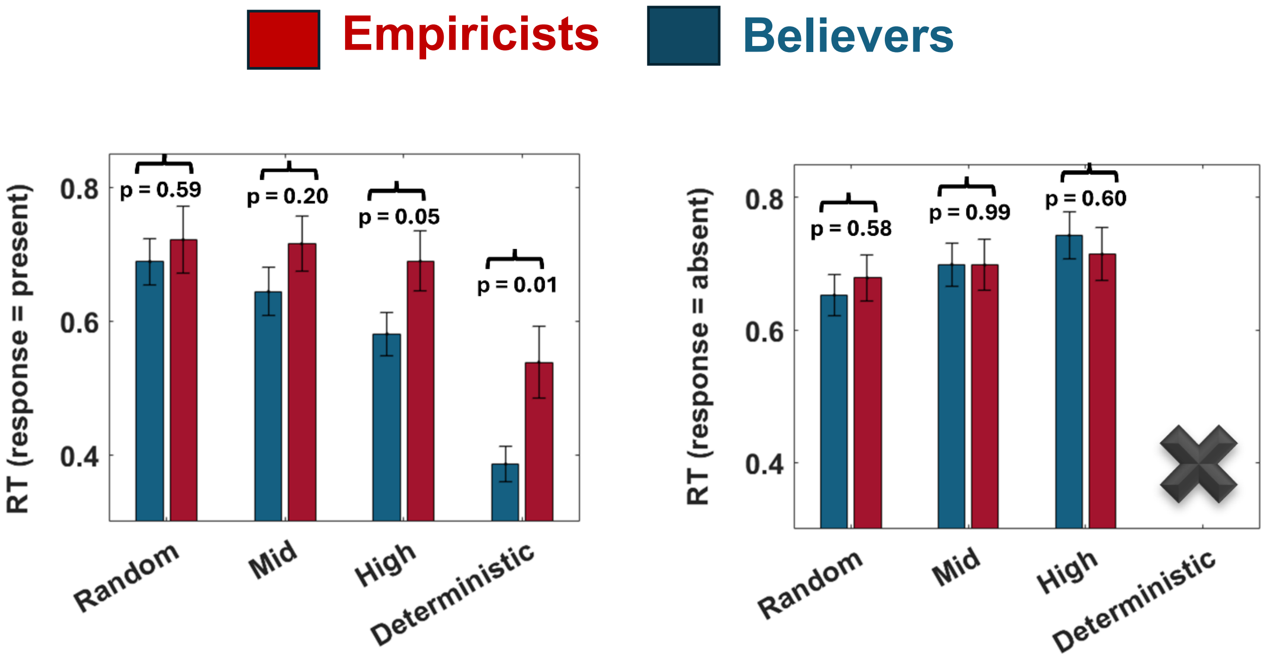

Figure S5 Reaction times (RTs) reveal group differences in prior congruency effects.

(A) For present responses, Empiricists showed faster RTs in the deterministic condition compared to all other conditions and also responded quicker in the high-predictive condition compared to the mid-predictive one. In contrast, Believers exhibited a progressive RT acceleration with increasing cue predictability. Believers were faster than Empiricists in both high-predictive and deterministic conditions.
(B) For absent responses, Empiricists responded slower in the high-predictive condition compared to the random one, while Believers showed progressively slower RTs as cue predictability increased. These findings highlight stronger prior congruency effects in Believers, reflected in faster responses for congruent choices and slower responses for incongruent ones.

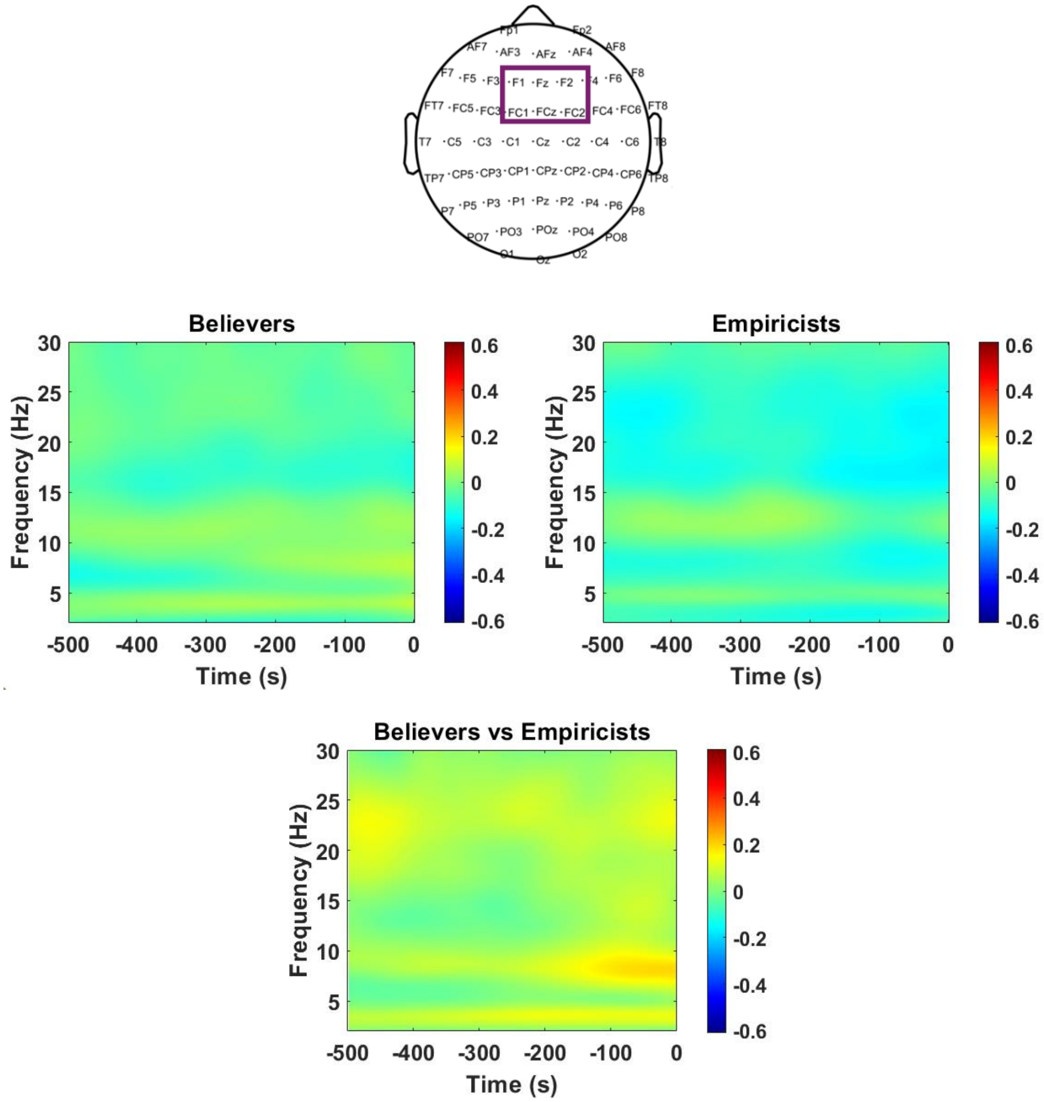

Figure S6. Time-frequency activity in the fronto-central electrodes in the random condition.

The cluster-based permutation analysis that compared the time-frequency activation in fronto-central electrodes between believers and empiricists under the condition where prior had random predictive power did not reveal any significant differences (p cluster = 0.1). This implies that activity levels in the random condition are comparable between the two groups of participants.

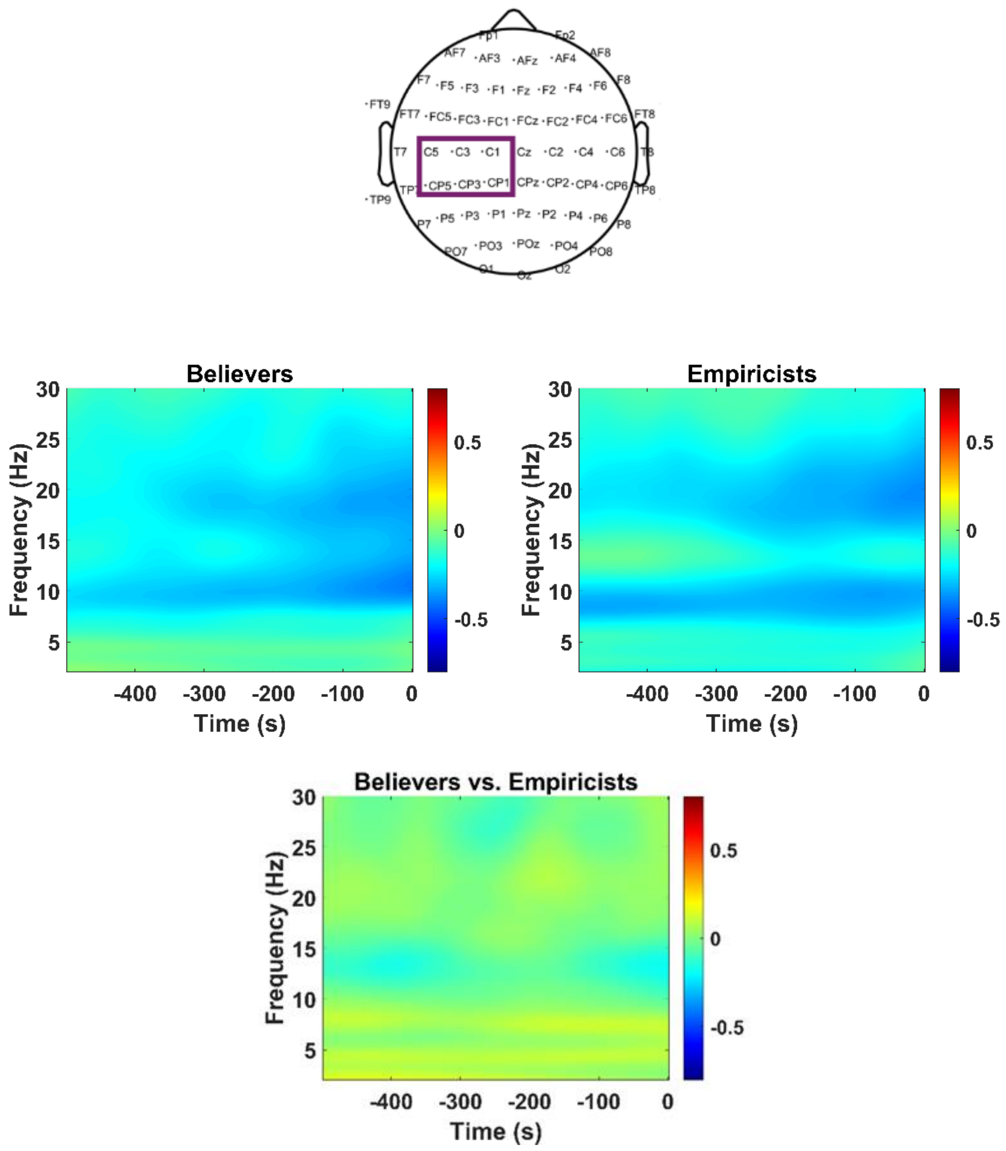

Figure S7. Time-frequency activity in the left-central electrodes in the random condition.

The cluster-based permutation analysis that compared the time-frequency activation in left-central electrodes between believers and empiricists under the condition where prior had random predictive power did not reveal any significant differences (p cluster = 0.23). This implies that activity levels in the random condition are comparable between the two groups of participants.

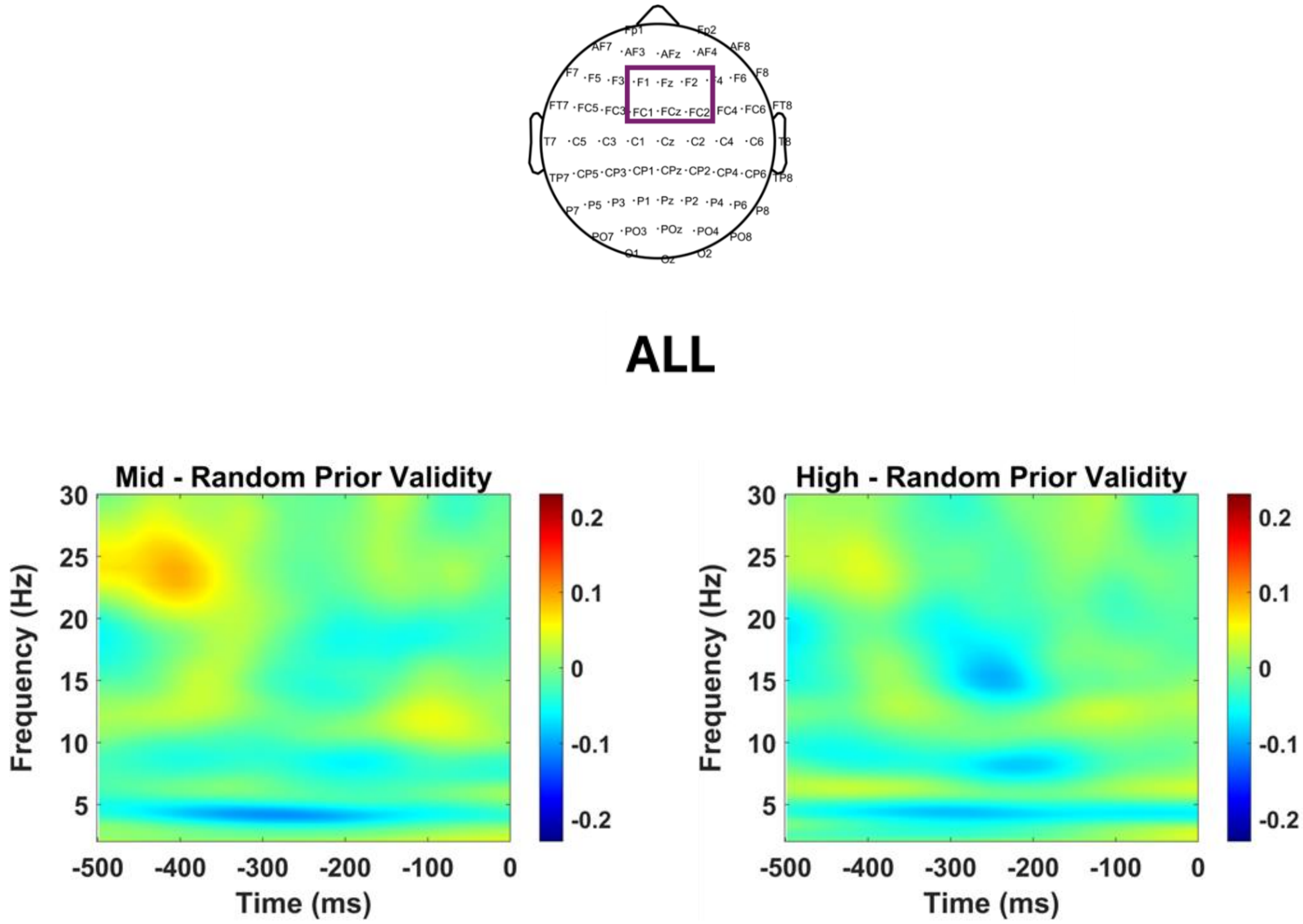

Figure S8. Time-frequency activity in the fronto-central in probabilistic condition in the whole sample.

No differences were observed in the probabilistic conditions when considering the entire sample (p mid – random = 0.16; p high – random = 0.23). This result is due to the fact that the pre-stimulus theta inactivation effect is present only in the believers' group, while no effect is observed in the empiricists group.

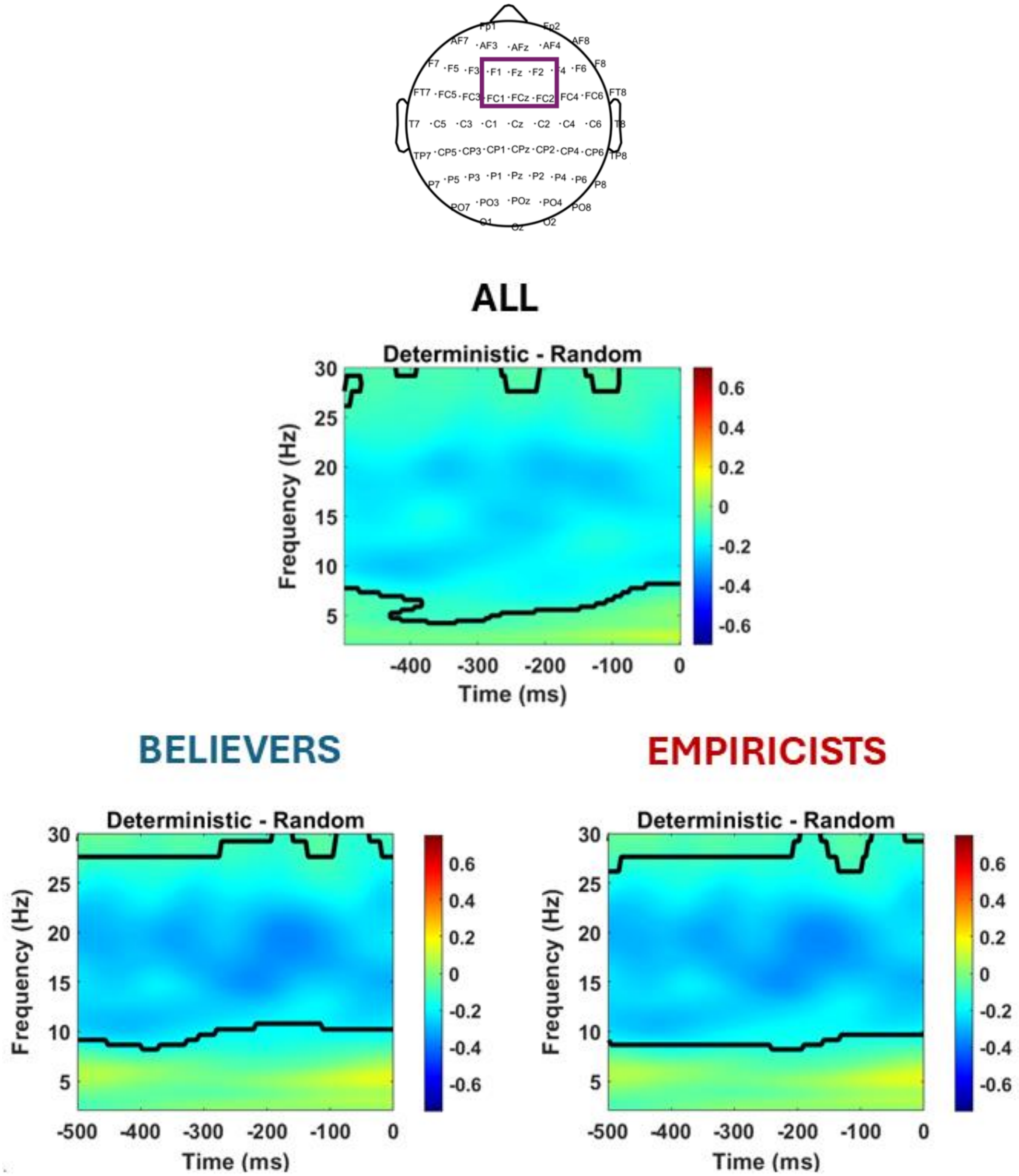

Figure S9. Time-frequency activity in the fronto-central electrodes in the deterministic vs. random condition.

The cluster-based permutation analysis, comparing time-frequency activation in fronto-central electrodes in believers and empiricists under conditions where prior information had deterministic versus random predictive power, revealed significant differences peaking in the beta band (p clusters < 0.01). As demonstrated in the main text, this strong beta desynchronization pattern originates from the left motor cortex, preparing for prior-congruent responses. However, these patterns are still captured by midfrontal electrodes, which are not far from the motor regions. Importantly, unlike the probabilistic settings, no differences emerged in the theta band. Additionally, there was no significant association between the hit rate shift between the deterministic and random conditions and the beta differences recorded in the fronto-central sensors (Spearman = 0.26, p > 0.05). This contrasts with the strong association found between beta synchronization recorded in left-central electrodes, indicating that the fronto-central effect could be due to a spatial smearing effect.

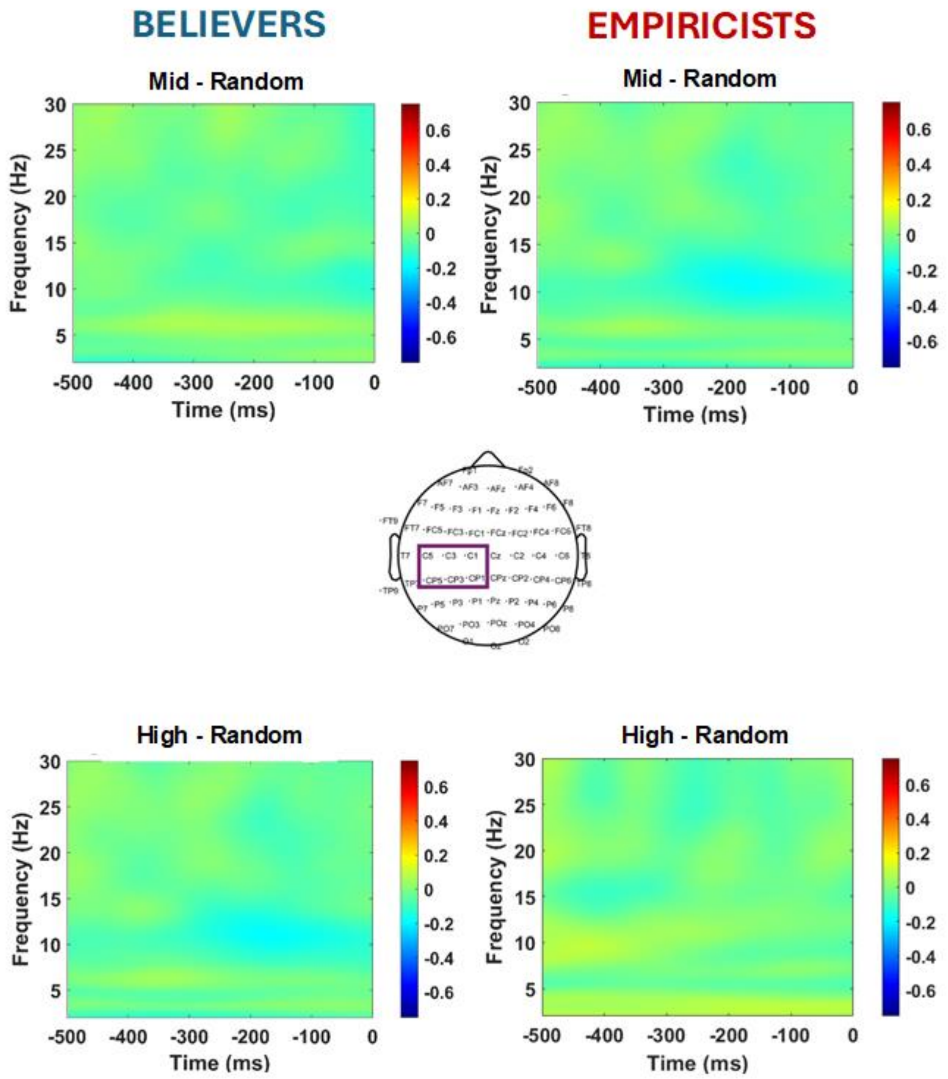

Figure S10. Time-frequency activity in the left-central electrodes in the mid vs. random condition and high vs. random condition.

The cluster-based permutation analysis, comparing time-frequency activation in left-central electrodes in believers and empiricists under conditions where prior information had mid/high versus random predictive power, did not reveal significant differences peaking in the beta band as showed in the deterministic condition (all p cluster > 0.19). We can speculate that, even though it is possible to prepare a motor response congruent with the information provided by the prior in conditions of mid and high prior validity, the probabilistic nature of the information, rather than deterministic, does not elicit a motor preparation effect that is discernible enough to be significant.

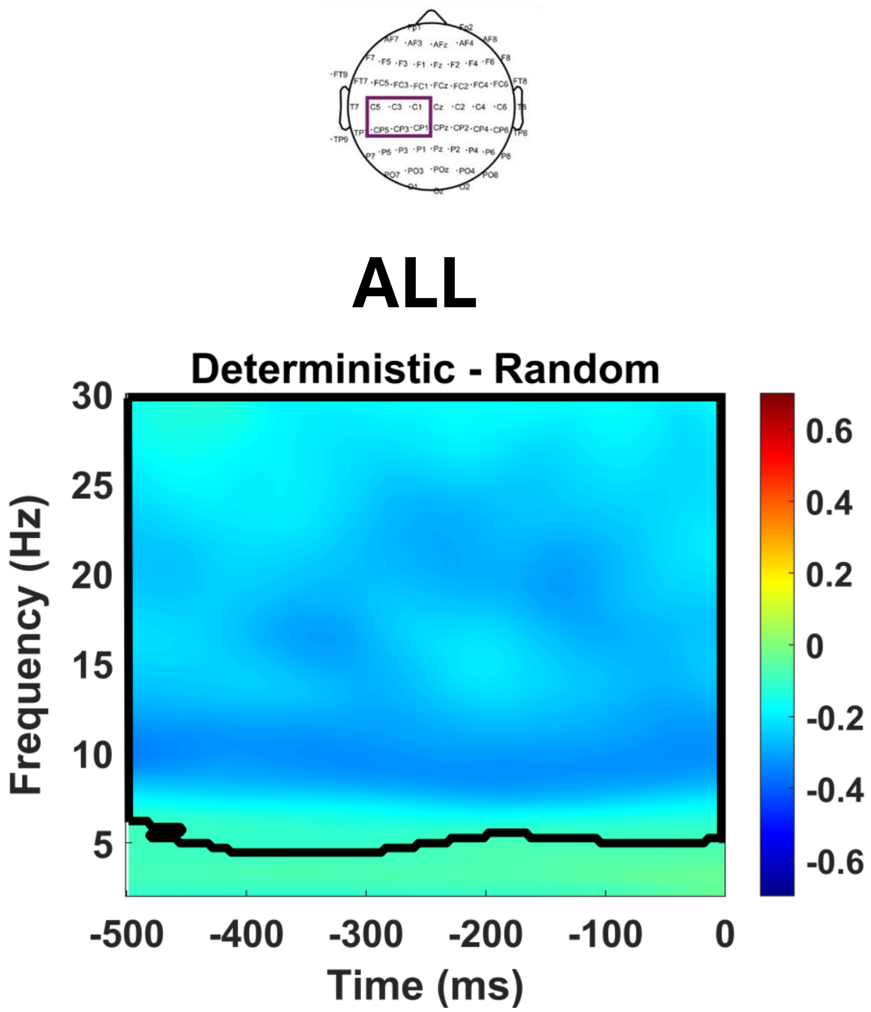

Figure S11. Time-frequency activity in the left-central electrodes in the deterministic - random condition.

The cluster covering the beta frequencies shows that, when considering the entire sample, there is a strong desynchronization in the deterministic condition compared to the random condition (p cluster < 0.01). Crucially, as shown in the main text, this effect is statistically stronger in the believers group compared to the empiricists group.

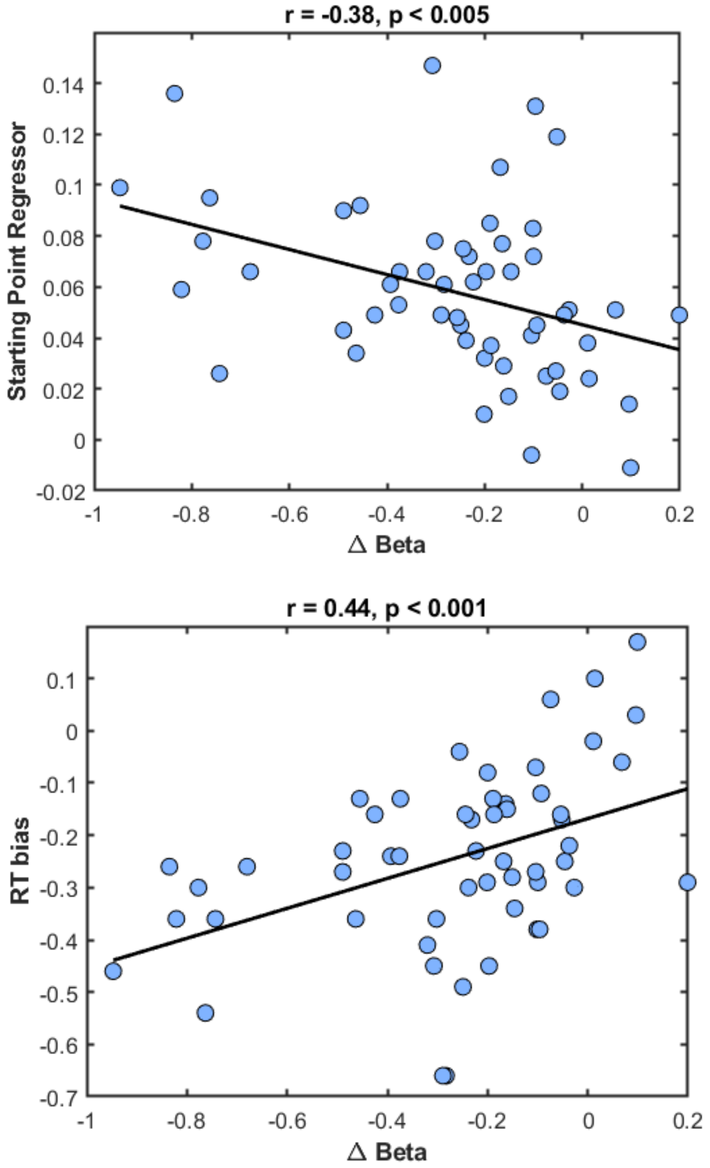

Figure S12. Relationship between Δ beta and starting point and RT bias.

In the figure above, we show the correlation between Δ beta and starting point regressor. The results indicate that participants who exhibit strong beta desynchronization in the left-central areas are those who show a marked increase in the starting point parameter. In contrast, participants who display an opposite pattern of synchronization maintain a consistent starting point (i.e., a consistent bias) in probabilistic conditions.

In the figure below, we show the correlation between Δ beta and RT bias. The results indicate that participants who exhibit strong beta desynchronization in the left-central areas are those who show a marked acceleration in the deterministic condition compared to the random condition. In contrast, participants who display an opposite pattern of synchronization maintain a consistent RT in both conditions.

Extracting believers and empiricists using raw behavioral data led to the same pattern as when using the starting point regression.

We employed an alternative method to split the data based on more direct and raw behavioral metrics in order to provide stronger confirmation of the findings presented in the main text. Specifically, we considered a straightforward measure of detection performance: the hit rate and false alarm rate. These two indices were combined into a single metric, the Response Bias Index, which we used to divide the sample into two subgroups through a median split analysis. This index was derived from:

- The difference between the HIT RATE in the "mid" condition and the "random" condition.
- The difference between the HIT RATE in the "high" condition and the "random" condition.
- The difference between the FALSE ALARM in the "mid" condition and the "random" condition.
- The difference between the FALSE ALARM in the "high" condition and the "random" condition.

$$\text{Responce bias index} = \text{mean}\left([HR_{mid,present} - HR_{random,present}],[HR_{high,present} - HR_{random,present}],[FA_{random,absent} - FA_{mid,absent}],[FA_{high,absent} - FA_{random,absent}]\right)$$

The rationale behind this approach is that a higher value of the Response Bias Index indicates a greater response bias in the participant. Specifically, participants exhibit a higher Hit Rate and False Alarm Rate as the probability of target presence increases. This method allows us to combine the behavioral conditions into a single, interpretable measure, while relying on a “rawer” behavioral index. We then applied a median-split analysis to this index and repeated the cluster-based analyses to determine whether the findings from the main text could be replicated. These findings included the absence of theta activation in the probabilistic conditions—specifically in the believers—and the pronounced beta synchronization in the left-central areas of the believers. As shown in the time-frequency map below, we successfully replicated both findings. Specifically, believers exhibited both a lack of activation in the probabilistic condition and a stronger desynchronization pattern in the left-central electrodes within the beta band compared to the empiricist group.

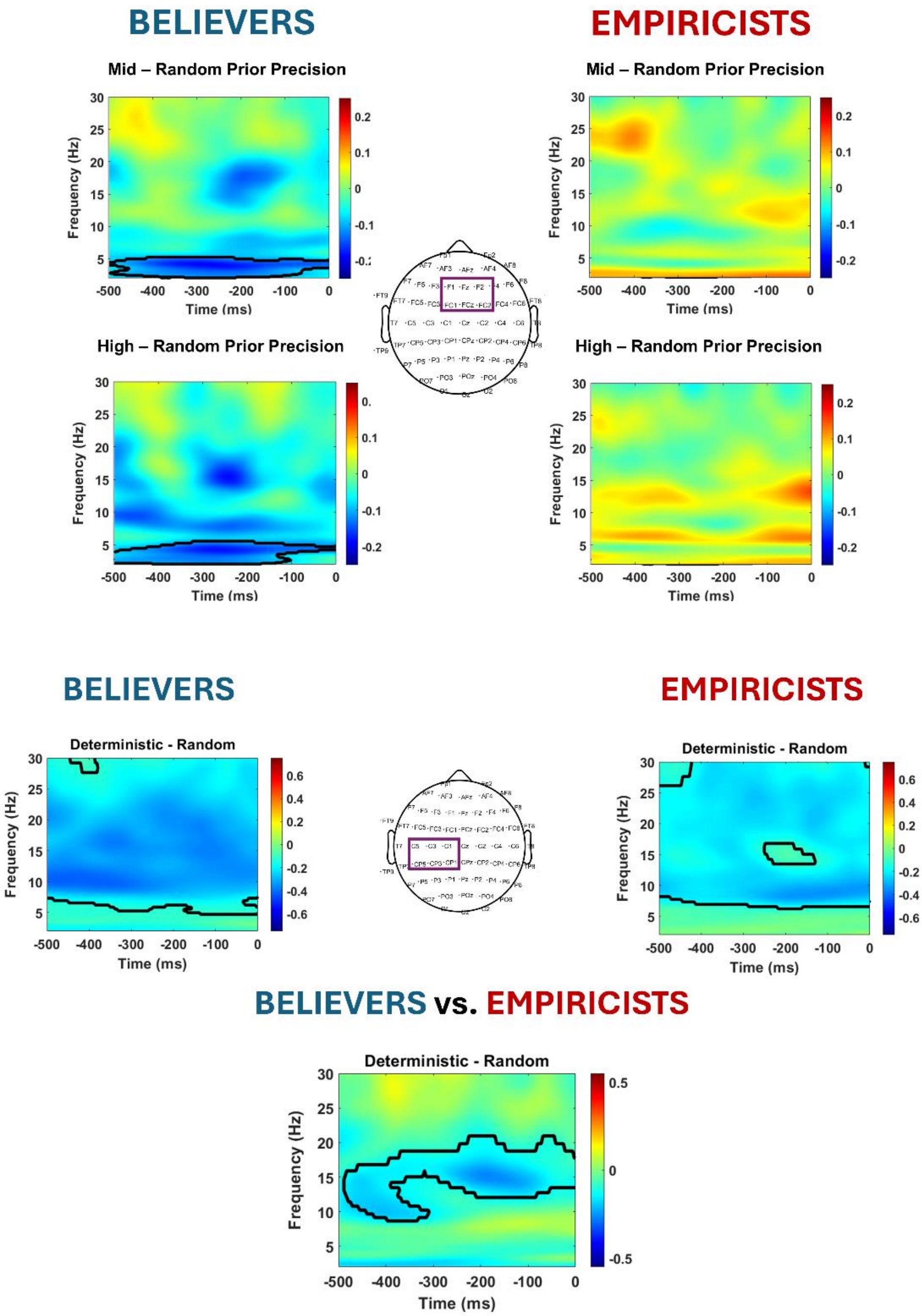

In addition, we performed an additional check by evaluating how the new division into believers and empiricists was associated with different values of the starting point regressor in the two groups. This analysis serves as further corroboration of the validity of the measure, which should show differentiation based on the division made according to hit rates and false alarms. The independent samples t-test revealed a significant effect, demonstrating that the groups extracted through the Response Bias Index showed a significantly different regression coefficient (starting point regressor $_{believers}$ = 0.08 ± 0.01, starting point regressor $_{empiricists}$ = 0.04 ± 0.01, t_{52} = 5.29, $p < 0.01$).

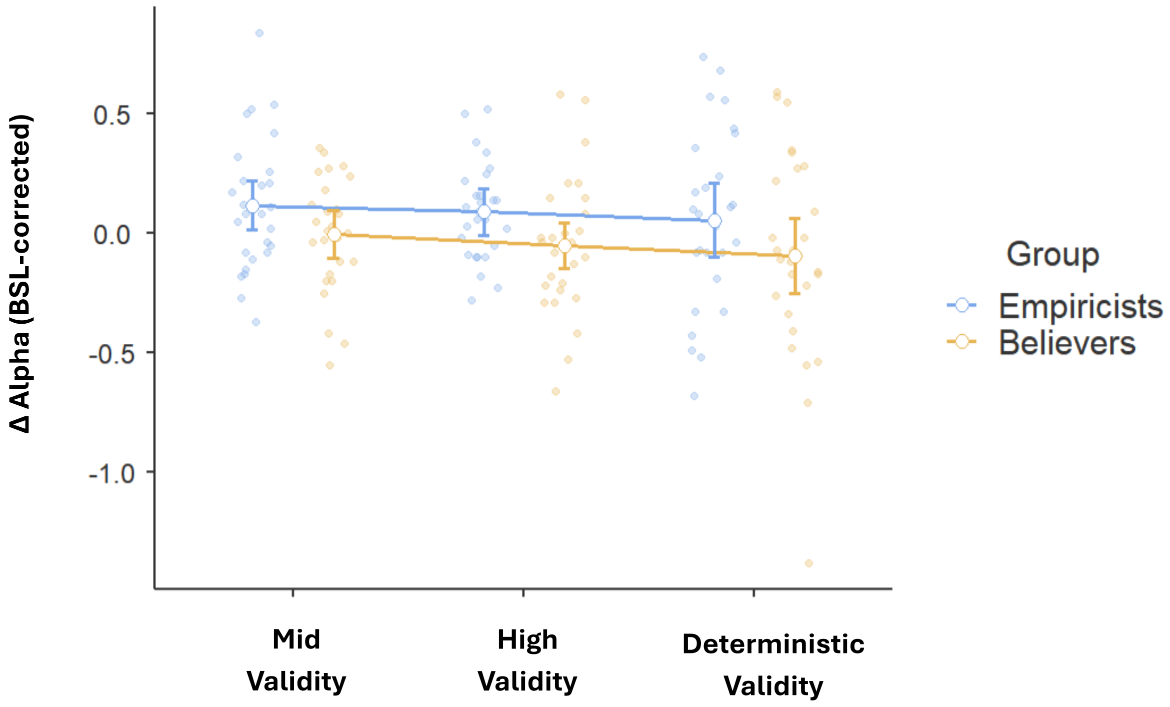

In previous studies (Tarasi et al., 2023, 2022), we distinguished between believers and empiricists by examining how posterior alpha oscillations changed after presenting a probabilistic prior. This method was effective as alpha oscillations are particularly responsive in situations in which different strategies have to be employed. For instance, in our previous paradigms, trials with a low target occurrence probability (33%) favored the adoption of a conservative criterion, while those with a high target occurrence probability (67%) favored a liberal criterion. Crucially, we found that alpha synchronized in conservative contexts but desynchronized in liberal ones. Importantly, Kloosterman et al. (2019) also observed a very similar pattern in contexts favoring conservative vs. liberal criteria.

In the current experimental protocol, given the graded nature of the prior manipulation towards liberal only cues, we did not expect alpha modulations to finely discern empiricists and believers. However, as a demonstration that our behavior-based determination of empiricist and believers groups tapped on the same alpha shift mechanism we tested whether the behavioral-driven definition of empiricist and believers groups could show a differential effect of alpha amplitude shift in the liberals vs the random condition at the group level. To this purpose, we ran an ANOVA having as dependent variable the random-corrected alpha amplitude (8-14 Hz) extracted from posterior electrodes including, *as between-subjects factor, the group (2 levels: empiricists and believers) and, as within-subject factor, the prior validity (3 levels: mid, high, and deterministic probability). The conducted analysis revealed the presence of a significant group effect ($F_{1,52} = 5.38$, $p = 0.02$), while both the cue factor as well as the cue* group interaction were not significant (all $F_{2,104} < 1.19$, all $p > 0.31$). Specifically, believers exhibited higher alpha desynchronization under conditions where the prior gains validity. This result demonstrates that, although the current experimental protocol was not designed to elicit a strong alpha-related effect, alpha oscillations still show a significant modulation associated with different cognitive styles which are differently modulated in the two groups. We also conducted the same analysis, this time using theta amplitude extracted from posterior regions as the dependent variable. The analysis included group as a between-subjects factor (2 levels: empiricists and believers) and prior validity as a within-subject factor (3 levels: mid, high, and deterministic probability). This analysis revealed no significant differences (all $F_{2,104} < 1.72$, all $p > 0.19$), supporting the conclusion that theta modulation is specifically related to fronto-central regions involved in cognitive control.*

Analysis of Temporal Stability in Behavioral and Neural Predictive Strategies

We conducted additional analyses to assess the presence of time-dependent effects on both behavioral and neural measures. To this end, we performed separate ANOVAs for hit rates, false alarms, frontocentral theta oscillations, and motor beta oscillations. Trials were divided into tertiles, and we analyzed behavioral performance and neural indices across these three tertiles to identify any effects attributable to elapsed time.

For hit rates, the ANOVA included the between-subjects factor group (2 levels: believers and empiricists) and the within-subjects factors cue (4 levels: random, mid, high, deterministic) and time (3 levels: first, second, and third tertile). The analysis revealed a significant cue × group interaction ($F_{3,156} = 7.71$, $p < 0.01$), while all other interactions were non-significant (all $F < 1.51$, all $p > 0.23$). For false alarms, the ANOVA followed a similar structure but reduced the cue factor to 3 levels (false alarms are not possible in the deterministic condition). Again, the ANOVA revealed a significant cue × group interaction ($F_{2,208} = 6.44$, $p < 0.01$), with all other interactions being non-significant (all $F < 1.53$, all $p > 0.19$). These results indicate that while differences in hit rates and false alarms depend on the cue and group (as previously reported in the main text), these measures are not influenced by the factor time. Thus, we can conclude that decision-making strategies in our sample exhibit strong temporal stability.

Turning to neural indices, we conducted two separate ANOVAs, one with frontocentral theta oscillations as the dependent variable and the other with left-central beta oscillations. In both ANOVAs, we included the between-subjects factor group and the within-subjects factors cue (3 levels: mid, high, deterministic—the random condition was used as baseline, consistent with the main text) and time. For frontocentral theta oscillations, the analysis revealed a significant cue × group interaction ($F_{2,208} = 3.18$, $p = 0.046$), while all other interactions were non-significant (all $F < 1.51$, all $p > 0.22$). Similarly, for left-central beta oscillations, the cue × group interaction ($F_{2,208} = 5.72$, $p < 0.01$) and the time x cue ($F_{2,208} = 2.92$, $p = 0.02$) were significant.

In summary, the neural results align with the behavioral findings: the predictive strategies adopted by participants remain stable over time, with consistent behavioral and neural patterns observed across the three tertiles analyzed.
